# Effectiveness of lay workers delivering behavioural activation for people with depression: systematic review and meta-analysis

**DOI:** 10.1192/bjo.2026.12018

**Published:** 2026-06-25

**Authors:** Shwikar Othman, Lauren Blekkenhorst, Asangaedem Akpan, Irene Ngune, Nilufeur McKay, Alison Kennedy, Elizabeth Armstrong, Michael Hunter, Kevin Murray, Mathew Coleman, Richard Gray, Emma Jones, Christopher Lee, Martin Jones

**Affiliations:** School of Nursing and Midwifery, Edith Cowan University, Perth, Western Australia, Australia; Nutrition and Health Innovation Research Institute, School of Medical and Health Sciences, Edith Cowan University, Perth, Western Australia, Australia; Department of Geriatric Medicine, Bunbury Regional Hospital, Bunbury, Western Australia, Australia; Deakin University, Melbourne, Victoria, Australia; Department of Rural Health South West, Edith Cowan University, South West Campus, Bunbury, Western Australia, Australia; Busselton Health Study Centre, Busselton Population Medical Research Institute (BPMRI), Busselton, Western Australia, Australia; School of Population and Global Health, The University of Western Australia, Perth, Western Australia, Australia; Albany Regional Hospital, Albany, Western Australia, Australia; Healthy People, Families and Communities, LaTrobe University, Melbourne, Victoria, Australia; Rural Clinical School, University of Western Australia, Geraldton, Western Australia, Australia; Connell School of Nursing, Boston College, Massachusetts, USA; School of Nursing and Midwifery, Edith Cowan University, South West Campus, Bunbury, Western Australia, Australia

**Keywords:** Behavioural activation, lay workers, depression, effectiveness, meta-analysis

## Abstract

**Background:**

Behavioural activation is effective for depression, but its effectiveness in treating adults with depression when delivered by lay workers remains unclear.

**Aims:**

To examine the effectiveness of behavioural activation delivered by lay workers, compared with any control group, in reducing depressive symptoms in adults.

**Method:**

This systematic review searched six databases from inception to January 2025, for randomised controlled trials (RCTs) comparing behavioural activation and any control conditions for individuals with depression when delivered by lay workers. Additional searches were conducted in the international trial registries and reference lists (PROSPERO registration CRD42024625620). Risk of bias was assessed using the Cochrane Collaboration’s Risk-of-Bias 2 tool. Random effects meta-analysis was conducted using the Metafor package in R.

**Results:**

Of 9614 initial studies, six RCTs met the inclusion criteria and were included. A total of 1118 participants in the intervention groups and 1596 in the control groups. The findings demonstrated a small but statistically significant effect in reducing depressive symptoms in favour of the intervention group (standardised mean difference: −0.28, 95% CI −0.46 to −0.09; *p* = 0.0029). However, the risk of bias was high across all studies, with substantial heterogeneity (*I* ^2^ = 76%).

**Conclusions:**

Evidence from this review and meta-analysis suggests that behavioural activation, when delivered by trained lay workers, may offer an effective approach for reducing depressive symptoms in adults, particularly in settings with limited access to specialist mental healthcare professionals. However, high risk of bias and heterogeneity of the included studies means that these findings should be interpreted with caution.

Depression is a common mental disorder that characterised by persistent low mood and anhedonia, often accompanied by changes in self-worth, concentration, energy, sleep, appetite and weight.^1^ It is highly prevalent, affects people across all age groups and is more prevalent in women compared with the general population.^2^ Depression carries significant personal, social and economic burden including increased expenditure on healthcare, reduced productivity and risk of depression complications, such as cardiovascular disease, social isolation, anxiety, diabetes, sleep disorder breathing and chronic obstructive lung disease.^3–5^

The first-line treatments for mild to moderate depression often include pharmacological and psychological interventions, alone or in combination, and the most commonly used psychological intervention is cognitive–behavioural therapy (CBT).^6^ CBT is an effective ‘talking’ therapy for treating depression, and requires intensive training, ongoing supervision and time commitments from trained and qualified mental health professionals.^7–9^ In Australia, access to CBT is particularly limited in rural and very remote communities, where shortages of trained mental health practitioners and geographic isolation restrict access to mental health services. These barriers contribute to delays in seeking care and greater unmet mental health needs, underscoring the need for more accessible treatment options.^10,11^

Behavioural activation has emerged as an alternative option to CBT, and is one of the psychological therapies recommended in several treatment guidelines for the treatment of subthreshold, mild or moderate depression in adults.^6,12,13^ Behavioural activation is a structured psychological intervention that focuses on increasing access to adaptive behaviours, reducing avoidance behaviours that inhibit access to positive reinforcement, and resolving barriers to activation.^14^ It is considered less complex than CBT and has demonstrated similar effectiveness in treating subthreshold, mild and moderate depression.^9^ Several recent meta-analyses have reported that behavioural activation is effective in reducing depressive symptoms and can be applied across various conditions and target populations.^15–19^ For example, a systematic review targeting adults with comorbid non-communicable diseases concluded that behavioural activation may be a viable option for this population, highlighting its simplicity, adaptability and relevance for individuals whose physical health conditions may limit more complex therapeutic modalities.^17^ These findings suggest that behavioural activation is an evidence-based intervention whose core behavioural principles, such as engagement in meaningful activities and reinforcement of positive behaviours, can be adapted across diverse contexts. In addition, a recent Cochrane review of 53 randomised controlled trials (RCTs) involving 5495 participants found moderate certainty evidence that behavioural activation had greater short-term efficacy compared with treatment as usual for adults with depression, and found no significant differences in efficacy between behavioural activation and CBT.^20^

Mood monitoring and activity scheduling are common behavioural activation techniques and have been shown to be effective in helping individuals identify patterns in their mood, increase engagement in rewarding activities and reduce depressive symptoms.^21^ These techniques are simple to teach and implement, and suit delivery by non-specialist providers.^21^ Utilising behavioural activation in preventive interventions to manage and control early depressive symptoms may help mitigate the individual, family and societal costs associated with the burden of depression. Limited access to mental health professionals has led to a growing interest in whether non-specialist workers, such as lay workers, can deliver low-intensity mental health interventions like behavioural activation.^22^ According to the World Health Organization (WHO), lay workers are community members who have received some training to promote health or to perform some healthcare services, but are not healthcare professionals.^23^ A previous Cochrane review, including 82 RCTs, examined the effect of interventions delivered by lay workers to improve maternal or child health or infectious disease management.^24^ The review concluded that lay workers offer promising benefits in promoting immunisation uptake and breastfeeding, improving tuberculosis treatment outcomes and reducing child morbidity and mortality compared with usual care. For other health issues (e.g. HIV/AIDS prevention, malaria control), evidence was insufficient to draw conclusions about the effects of interventions delivered by lay workers.^24^ Although the use of lay workers in the before mentioned health outcomes has been explored, there is currently no systematic reviews examining the effectiveness of behavioural activation when delivered by lay workers to adults with depression. Therefore, the aim of this systematic review was to examine the effectiveness of lay worker-delivered behavioural activation in reducing depressive symptoms in adults with depression.

In this review, we will use the definitions of non-specialist workers and lay workers as provided by the WHO and Uphoff et al.^20^ The term ‘non-specialist worker’ is heterogeneous, comprising two distinct groups: health professionals without specialist mental health training and lay workers with no clinical background.^20,25^

## Aims

In this review, we aimed to examine the effectiveness of behavioural activation in reducing depressive symptoms in adults when delivered by lay workers compared with any control group.

## Method

The systematic review methodology was reported in accordance with the Preferred Reporting Items for Systematic Reviews and Meta-Analyses (PRISMA) guidelines,^26^ and followed a prespecified protocol registered in the International Prospective Register of Systematic Reviews protocol (PROSPERO identifier: CRD42024625620). The protocol was registered on 20 December 2024.

### Inclusion criteria

#### Population

Adults aged ≥18 years diagnosed with depression or who report symptoms of mild to severe depression were included.

#### Intervention

The review focused on behavioural activation delivered by lay workers as the intervention. All RCTs evaluating treatment approaches for depression that were clearly described as ‘behavioural activation’, or treatments defined using the key components of behavioural activation, such as mood monitoring and activity scheduling, were included. Treatment approaches that incorporated elements of behavioural therapy, such as CBT or problem-solving therapy, were excluded.

#### Mode of delivery of behavioural activation

The mode of behavioural activation delivery could include face-to-face, online or group format by lay workers, including non-professional therapists, lay counsellors, lay workers, lay health workers, non-credential workers, voluntary workers or non-specialist persons. Lay counsellors who used psychological therapies targeting individuals or groups were eligible for inclusion without giving regard to the number of sessions. If behavioural activation was delivered by lay workers under the supervision of a therapist or psychiatrist, the study was included. However, if the lay worker was part of a team led by a psychiatrist or a psychotherapist who delivered the behavioural activation, the study was excluded.

#### Comparator intervention

Comparator interventions included waiting lists for treatment, usual care, comparative depression treatments or no treatments.

#### Outcome

The main outcome measure was the change in depressive symptoms, assessed by using scores from standard self-/clinician-/researcher-administered clinical rating scales from baseline to follow-up. Primary outcome measures included common depression scales such as the Patient Health Questionnaire-9 (PHQ-9)^27^ or Modified Beck Depression Inventory Version II (BDI-II).^28^ If a study included multiple scales for the same outcome, only the most used scale was selected.

#### Types of studies

Only RCTs were included. Other studies that were original and not RCT were excluded. All qualitative studies, study protocols, review protocols, commentaries, reports and systematic reviews were excluded. Other excluded studies include abstracts for conference proceedings or abstract with no full text or detailed results. Studies including participants with bipolar depression were also excluded.

#### Setting

This review included trials conducted in primary, secondary or community settings.

### Search strategy

This systematic review adhered to the PRISMA guidelines.^26^ Six databases were used to identify RCTs of behavioural activation for depression in adults, and included Ovid Medline, Ovid Embase, Ovid Emcare, Ovid PsycINFO, CINAHL and the Cochrane Library. Searches were also conducted in international trial registries, including the WHO International Clinical Trials Registry, the Australian New Zealand Clinical Trials Registry and ClinicalTrials.gov.

Reference lists of the included studies were checked to ensure all RCT trials were captured from the original electronic database searches. The first ten pages of Google Scholar were also searched. No restrictions on date, language or publication status was applied; however, unpublished data were not considered. All database searches were conducted between November and January 2025 (Supplementary Appendix 1: All databases search strategy).

### Data collection and analysis

#### Study selection

All database search results were imported into Endnote reference management software version 2025 for Windows (The EndNote Team, Clarivate, London, UK; https://supportcenter.clarivate.com/s/article/Citing-the-EndNote-program-as-a-reference) and then uploaded into the Covidence systematic review management software for Windows (Veritas Health Innovation, Melbourne, Australia; www.covidence.org), where duplicates were identified and removed electronically. Two authors (A.A., M.J., I.N., S.O., N.M. or L.B.) independently screened each title and abstract of all records identified through the search strategy. Full-text articles of all identified trials were retrieved, and two authors (A.A., M.J., I.N., S.O., N.M. or L.B.) independently assessed the studies for eligibility based on the inclusion criteria. All disagreements for both title and abstract as well as full-text screening were resolved through team discussion, to reach a consensus based on percentages. Reasons for exclusion were recorded (Supplementary Appendix 2: list of excluded studies). A PRISMA flow diagram was used to present the study selection process.^26^

#### Interrater reliability agreement assessment

Interrater agreement was evaluated using both the proportion of observed agreement and Cohen’s *κ* coefficient.^29^ For title and abstract screening, reviewers demonstrated consistently high raw agreement (83–99%), but because ‘yes’ ratings were very rare, Cohen’s *κ* was reduced, with most pairs showing only slight to fair agreement, and one pair showed moderate agreement (*κ* = 0.53), some pairs had non-computable *κ*-values because of all ‘no’ ratings. For full-text screening, observed agreement was generally high, but the predominance of ‘exclude’ ratings limited the interpretability of Cohen’s *κ*. Among pairs with sufficient variation, agreement ranged from poor to moderate, with the highest at *κ* = 0.59. Overall, the reviewers were largely consistent in their decisions, indicating that Cohen’s *κ* underestimated the true level of agreement.

#### Data extraction and management

Data extraction was conducted independently by two review authors working in pairs (A.A., I.N., S.O., N.M. and L.B.). Each included study was independently extracted by two review authors, using a standardised data extraction sheet. The data extraction sheet was adapted from a template used in a previous study by the review authors.^17,19^ To ensure its suitability for the current review, the template was pre-tested for clarity and consistency. The extracted data included study characteristics such as study population, setting, recruitment method, treatment, outcome measures, randomisation, sample size calculation, measurements, effect size, risk of harm and reported adverse effect. All extracted data were compiled and cross-checked by a review author (S.O.) for completeness and accuracy. This additional verification step was undertaken to enhance consistency across the authors, minimise errors and ensure the overall reliability and transparency of the extracted data. Data extraction started on 4 March 2025.

#### Critical appraisal and risk of bias

Three review authors (S.O., M.J. and L.B.) independently assessed the risk of bias of each RCT by using version 2 of the Cochrane Risk-of-Bias Tool for randomised trials.^30^ Consensus was reached through discussion among the review authors. Data from the risk of bias assessment were entered into robvis, a risk-of-bias data visualisation tool, to generate a summary figure.^31^ Risk-of-bias assessments were used to critique the research evidence, but not as an exclusion criterion. The risk of bias across all domains was summarised to produce an overall risk of bias for each RCT.

#### Data synthesis and meta-analysis

The review included a narrative synthesis of the findings from the included studies. To estimate the overall effect of behavioural activation delivered by lay workers compared with any control group in reducing depressive symptoms in adults, a meta-analysis was conducted. The analysis focused on synthesising evidence on the effectiveness of behavioural activation interventions delivered by lay workers versus treatment as usual or enhanced usual care. A random-effects meta-analysis using inverse variance weighting was conducted to account for variability across studies. The results were reported as pooled effect sizes expressed as standardised mean difference (SMD) with corresponding 95% confidence intervals. Negative SMD values indicated a reduction in depressive symptoms and therefore favoured behavioural activation compared with control conditions. Statistical heterogeneity among the studies was assessed using the *I* ^2^-statistic, and findings were visually presented in a forest plot. All analyses were conducted in R for Windows, using the meta and metafor packages (R Core Team, R Foundation for Statistical Computing, Vienna, Austria; https://www.r-project.org/). Subgroup analysis was conducted to explore potential sources of heterogeneity in the pooled estimate of the effect of behavioural activation on depressive symptoms, based on comparator type (enhanced usual care versus active psychoeducation).

The Grading of Recommendations Assessment, Development and Evaluation (GRADE) approach was used to assess the certainty of evidence for the outcome. The GRADE framework considers five domains: risk of bias, inconsistency, indirectness, imprecision and publication bias. Certainty was rated as high, moderate, low or very low. GRADE assessments were performed using GRADEpro GDT software for Windows (McMaster University and Evidence Prime, Hamilton, Canada; gradepro.org).

## Results

### Study selection

**Fig. 1:**
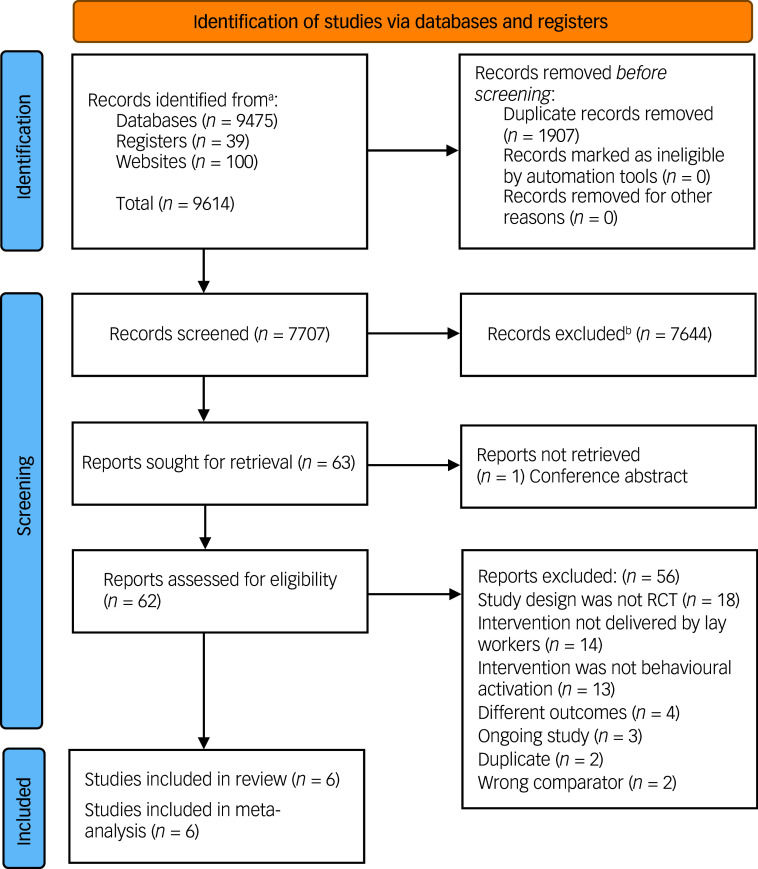
Preferred Reporting Items for Systematic Reviews and Meta-Analyses flowchart. Source: Page et al^26^. This work is licensed under CC BY 4.0. To view a copy of this license, visit https://creativecommons.org/licenses/by/4.0/. RCT, randomised controlled trial. a. Consider, if feasible to do so, reporting the number of records identified from each database or register searched (rather than the total number across all databases/registers). b. If automation tools were used, indicate how many records were excluded by a human and how many were excluded by automation tools.

A total of 9614 citation were identified and uploaded into Covidence systematic review software. A total of 1905 citation were removed electronically as duplicate. The title and abstracts of the remaining 7707 records were screened against the inclusion criteria. Of these 7644 records were excluded as irrelevant. The remaining 63 articles were eligible for full-text screening. Fifty-seven studies were excluded for the following reasons: study design was not RCT (*n* = 18), intervention was not delivered by lay workers (*n* = 14), the intervention was not behavioural activation (*n* = 13), different outcomes (*n* = 4), ongoing study (*n* = 3), duplicate (*n* = 2), wrong comparator (*n* = 2) and abstract with no full text available (*n* = 1). Six studies met the inclusion criteria and were included in the review and meta-analysis (Fig. 1). A list of excluded studies is presented in a Microsoft Excel sheet available in the Supplementary Material.

### Description of the included studies

Six studies met the inclusion criteria and were included.^32–37^ These studies were conducted between 2015 and 2024 across various countries, including China, India, Indonesia, Pakistan, the USA and Hong Kong (Table 1).

**Table 1:** Study characteristics

Study/country	Study design	Study aim	Inclusion criteria	Exclusion criteria	Population characteristics	Recruitment settings	Specific training provided for lay workers? By whom?
Au^33^ China	RCT	To test a model of telephone-assisted behavioural activation intervention for Chinese dementia caregivers, to reduce the level of depressive symptoms among these caregivers	Caregivers were aged ≥25 years and must have been caring for a care recipient diagnosed with Alzheimer’s for at least 3 months; they were the primary caregiver and a spouse/kin	Participants who showed signs of severe intellectual deficits, exhibited evidence of psychotic disorders or could not read or speak fluent Chinese/Cantonese were excluded	49 participants from the PsychED plus behavioural activation group, and 44 participants from PsychED only (control group). The majority lived with the care recipient (68.18–69.39%) and were female caregivers; most caregivers were either children of the care recipient (65.30–68.20%) or spouses (22.40–27.30%). The average hours the caregivers spent caregiving for their relative was 9.37–11.42 h a day	Usual residences	They completed a 42 h course and had received 20 h of group training led by a social worker and a clinical psychologist on behavioural activation in the context of the coping with caregiving programme. Each paraprofessional worker was tested on a mock case before delivering the programme to actual caregivers. Ongoing weekly supervision was provided by the clinical psychologist and social worker
Patel et al^36^ India	RCT	To assess the effectiveness and cost-effectiveness of a brief psychological treatment (HAP) for delivery by lay counsellors to patients with moderately severe to severe depression in primary healthcare settings	PHQ-9 score >14; 18–65 years, who had a probable diagnosis of moderately severe to severe depression	Pregnant women or patients needing urgent medical attention or were unable to communicate clearly were excluded	Total participants (*n* = 495 randomised; intervention *n* = 247; control *n* = 248) Intervention: 42.4 years old (*n* = 188, 76% female); control 42.6 years old (*n* = 191, 77% female). The modal patient was a married woman in her early forties with moderately severe depression, with a primary school education, who was not employed outside the home	Primary healthcare centres	An international expert in behavioural activation trained and supervised five local specialists, who then trained and supervised lay counsellors. The lay counsellors underwent a 3 week participatory workshop on the healthy activity programme and alcohol counselling, followed by a 6-month internship delivering treatment to eligible patients in primary healthcare clinics, combined with peer-led group supervision. Eleven counsellors who met competency standards through role-plays and therapy quality measures participated in the trial. They received weekly peer-led supervision (in groups of four to six) and individual supervision twice monthly
Arjadi et al^32^ Indonesia	RCT	To examine the efficacy of GAF-ID (internet-based behavioural activation with lay counsellor support) compared with minimal online psychoeducation delivered without support for depression	Aged ≥16 years, score ≥10 on PHQ-9, diagnosed with major depression or persistent depressive disorder based on the SCID-5, were proficient in Bahasa Indonesia, and could use the internet	Current substance use disorder, current or previous manic or hypomanic episodes or psychotic disorder, and acute suicidality and receiving weekly psychological interventions	The study includes two groups: GAF-ID (*n* = 159) and online psychoeducation (*n* = 154) Mean age, years: GAF-ID 24–45 (s.d. 4.93); online psychoeducation 24–52 (s.d. 5.22) Education level: Bachelor’s degree: GAF-ID (*n* = 76, 48%); online psychoeducation (*n* = 73, 47%) Occupation: Unemployed: GAF-ID (*n* =18, 11%); online psychoeducation (*n* =6, 4%) Private employee: GAF-ID (*n* =56, 35%); online psychoeducation (*n* =48, 31%) Student: GAF-ID (*n* =57, 36%); online psychoeducation (*n* =63, 41%)	Community dwellings and residences	Lay counsellors underwent 2 days of intensive training on the Internet-based behavioural activation programme, which included role-playing exercises and handling technical issues, such as potential platform problems, managing participants with low motivation, and monitoring suicidality or serious deterioration during the intervention. Lay counsellors received printed training modules for ongoing reference throughout the trial. To maintain treatment integrity and consistency, additional resources were provided including a treatment guidance protocol, a weekly support checklist, a participant progress report template and regular supervision by a clinical psychologist
Sikander et al^37^ Pakistan	Cluster RCT	To evaluate the effectiveness and cost-effectiveness of the adapted THPP intervention compared with enhanced usual care in two diverse rural and urban contexts in South Asia (Pakistan and India)	Women aged ≥18 years in their third trimester, registered with local lady health workers and intending to stay in their village cluster for at least a year post-enrolment. Participants scored >10 PHQ-9 at the baseline	Inability to speak Urdu, Hindu or Potohari and require immediate medical or psychiatric in-patient care	Total number of participants were *n* = 224 for the intervention, and *n* = 211 for the control. Average age was 26.80 (s.d. 4.60) years for the THPP and enhanced usual care, and 27.28 (s.d. 4.97) years for enhanced usual care only. All women were married. Highest education attained was secondary. Women were in a low socioeconomic region, exposed to poverty, high illiteracy rates and high fertility rates (3.8 births per woman)	Rural, village-based setting	Lay workers received brief classroom training, followed by ongoing group training and field supervision from non-mental health specialist local THPP trainers, who were themselves supervised by a specialist therapist in a cascade model of training and supervision. Trainer competency was assessed using an ENACT rating scale, both immediately and after training, and at 6 months. Lay workers scoring ≥70% were deemed competent and assigned to support women with depression
Choi et al^34^ USA	RCT	To test the acceptability and effectiveness of lay-coach-facilitated, video conferenced, short-term behavioural activation intervention for improving social connectedness among homebound older adults	Participant confirmation of loneliness scale score ≥6, no to mid depressive symptoms (PHQ-9 <10) and willingness to participate, aged ≥50 years in Texas and ≥60 years New Hampshire, in accordance with home-delivered meals programmes’ eligibility criteria	Probable cognitive impairment (score >9 on the Blessed Orientation, Memory, and Concentration Test), substance misuse disorders, active suicide ideation	Total number of participants *N* = 89 (tele-behavioural activation *n* = 43; tele-friendly visit *n* = 46) Average age 74 years, 62% female, 18% non-Black, 15% Hispanic, 68% lived alone, 83% had a household income <$29 000. No difference in tele-behavioural activation versus tele-friendly visit	Community	Both behavioural activation and friendly visit interventionists were trained by the first and second authors and had ongoing supervision throughout the study
Kwok et al^35^ Hong Kong	Three-arm RCT	To compare the effects of layperson-delivered, telephone-based behavioural activation and mindfulness interventions vs telephone-based befriending on loneliness among at-risk older adults	Age ≥65 years, feelings of loneliness as indicated by a score of ≥6 on the three-item UCLA Loneliness Scale), with a monthly household income below US $577, and proficiency of Cantonese via telephone	Cognitive impairment, psychiatric disorders, limited access to the internet at home, and engagement in regular mindfulness or related mind–body practices at baseline (more than two times in each week)	4152 older adults were screened. Among 1917 eligible older adults, 1151 consented to participate (consent rate, 60.0%) and were randomised into the behavioural activation (*n* = 335), mindfulness (*n* = 460) and befriending (*n* = 356) groups. The mean age of participants was 76.6 (s.d. 7.8) years; 843 (73.2%) were female and 308 (26.8%) were male. The majority of participants were widowed or divorced (*n* =932, 81.0%), had primary education or below (*n* =782, 67.9%) and were living with three or more chronic diseases (*n* =505, 43.9%)	Public housing estates primarily catering to solo-living older adults, community centres for older adults, community centres for older adults and older adult academies	The training for peer counsellors involved six 2 h face-to-face weekly sessions conducted in small groups (four to six participants) led by an experienced social worker and a research assistant. The training sessions included lectures, practice sessions and role-plays that were developed by the research team

RCT, randomised controlled trial; PsychED, psychoeducation; HAP, Healthy Activity Program; PHQ-9, Patient Health Questionnaire-9; GAF-ID, Guided Act and Feel Indonesia; THPP, Thinking Healthy Programme peer-delivered; ENACT, ENhancing Assessment of Common Therapeutic factors rating scale; UCLA, University of California, Los Angeles.

These six included studies assessed the effectiveness of behavioural activation on depressive symptoms when delivered by lay workers. In terms of study design, four studies were two-arm RCTs,^32–34,36^ one study was a three-arm RCT^35^ and one was a cluster RCT (Table 1).^37^

### Study setting

Participants were recruited from various settings, including community-based settings,^34^ their usual residences^33^ and community dwellings.^32^ Recruitment also took place through primary healthcare centres^36^ and in rural, village-based locations.^37^ Additional settings included public housing estates primarily catering to solo-living older adults, community centres for older adults and older adult academies (Table 1).^35^

### Participants

The inclusion criteria across the included studies varied based on the study aim and target populations. Five studies included adults aged 18 years and older with a diagnosis of depression or reported symptoms of mild to severe depression, except for one study that recruited participants aged 16 years and older.^32^ Two trials recruited older adults aged 50 years and older^34^ or 65 years and older.^35^ Across all included studies, a total of 1118 participants were recruited into the intervention groups, and 1596 into the control groups (Table 2).

**Table 2:** Intervention and control group characteristics

Study/country	Intervention versus control allocated	Comparators	Number of behavioural activation sessions	Frequency of behavioural activation sessions	Duration of each behavioural activation session	Depression measure^a^	Timeline	Reported harms (behavioural activation/control)
Au^33^ China	(*n* = 51 *v*. *n* = 45) Attrition rate 3.1% (total enrolled *n* = 96, and total completed the study *n* = 93)	PsychEd-behavioural activation for the intervention group, and PsychEd for the control group (Telephone)	8 sessions bi-weekly for 4 months	Bi-weekly for 4 months	25–30 min	The Center for Epidemiologic Studies–Depression Scale	Time point 1 – baseline Time point 2 – post general PsychED programme after completing week 4 Time point 3 – within a week post-intervention (completion)	None reported
Patel et al^36^ India	(*n* = 247 *v*. *n* = 248) Attrition rate 5.8% (total enrolled *n* = 495, and total completed at 3 month follow-up *n* = 466)	Enhanced usual care plus HAP for the intervention group, and enhanced usual care for the control group (Face to face)	6–8 sessions	Weekly for 3 months	30–40 min	Modified Beck Depression Inventory version II Remission from depression as defined by a PHQ-9 score of <10, both assessed 3 months after enrolment	3 months	Serious adverse events and prescription of antidepressant medications were infrequent and did not differ between treatments. Any serious adverse event: *n* = 9 (4%) in the intervention group versus *n* = 10 (4%) in the control group, *p* = 1.00; deaths: *n* = 2 (1%) *v*. *n* = 0, *p* = 0.24; suicide attempts: *n* = 4 (2%) *v*. *n* = 3 (1%), *p* = 0.72; unplanned admissions to hospital: *n* = 3 (1%) *v*. *n* = 7 (3%), *p* = 0.34)
Arjadi et al^32^ Indonesia	(*n* = 159 *v*. *n* = 154) Attrition rate 15.3% (total enrolled *n* = 313, and total completed the 10 week assessment *n* = 265)	Behavioural activation intervention in the GAF-ID group. Online psychoeducation for the control group (Online)	8 sessions	Weekly for 10 weeks	30–45 min	PHQ-9	All participants completed the same self-report assessments at baseline, every 2 weeks until post-test assessment at week 10 and 3 and 6 month follow-up	15% (*n* = 47) of total participants reported increased suicide scores from baseline, but no serious deterioration was observed. Clinical assessments found no acute suicidality, so no referrals were made, and no adverse events were reported in either group
Sikander et al^37^ Pakistan	(*n* = 283 *v*. *n* = 287) Attrition rate 20% (total enrolled *n* = 570, and total completed 6 months of follow-up *n* = 456)	The adapted THPP plus enhanced usual care for the intervention group, and enhanced usual care for the control group (Face to face)	14 sessions (10 individual and 4 group)	14 sessions, from the third trimester to 6 months after childbirth	30–45 min	PHQ-9 (score >10 at baseline indicates depression; score ≤5 at 3 and 6 months indicates remission)	Baseline (third trimester), 3 months postpartum, 6 months postpartum	Overall, 43 (15%) women in the intervention group and 47 (16%) women in the control group had at least one serious adverse event, which were evenly distributed between the groups. The most common serious adverse events were death of the child, hospital admissions (mainly of the child) and experience of physical violence; however, there was no evidence of any difference between the groups
Choi et al^34^ USA	(*n* = 43 *v*. *n* = 46) Attrition rate 8.9% (total enrolled *n* = 89, and total completed 6 week follow-up *n* = 81)	Behavioural activation for the intervention group and friendly visit for the control group (Video conference)	5 sessions	Weekly	60 min	PHQ-9	Baseline, 6 weeks and again 12 weeks	No harms reported; outcome effect size reported for social interaction (DSSI-I) 8.8 (s.d. 1.6), loneliness (PrROMIS-L) 17.9 (s.d. 6.2), satisfaction with social support (DSSI-S) 14.4 (s.d. 3.0) and disability (WHODAS) 15.6 (s.d. 6.5)
Kwok et al^35, b^ Hong Kong	(*n* = 335 behavioural activation, *n* = 460 mindfulness, *n* = 356 befriending) ^c^Attrition rate 45.2% (total enrolled *n* = 1151, and total completed 1 month follow-up *n* = 631)	Three arms (tele-behavioural activation, mindfulness and befriending) (Telephone)	8 sessions	Bi-weekly for 6 months	30 min	The primary outcome was loneliness measured with the revised UCLA Loneliness Scale (Chinese version) and the De Jong Gierveld Loneliness Scale (Chinese). PHQ-9 (Chinese)	Baseline, 1 and 3 months	None from the behavioural activation group. Two participants from the mindfulness group died during the study period. However, these deaths were determined to be unrelated to the trial intervention because participants had unrestricted access to their usual healthcare and were not subjected to any lifestyle restrictions

PsychED, psychoeducation; HAP, Healthy Activity Program; PHQ-9, Patient Health Questionnaire-9; GAF-ID, Guided Act and Feel Indonesia; THPP, Thinking Healthy Programme peer-delivered; DSSI-I, Social Interaction Subscale of the Duke Social Support Index; DSSI-S, Social Satisfaction Subscale of the Duke Social Support Index; WHODAS, World Health Organization Disability Assessment Schedule; PROMIS-L, Patient-Reported Outcomes Measurement Information System, Social Isolation Scale; UCLA, University of California, Los Angeles.

a. Depression measure used for data analysis was the PHQ-9.

b. Kwok et al^35^ used three comparators; only the behavioural activation and mindfulness data were included in the meta-analysis.

c. A total of 1151 participants were enrolled, and 631 completed the 1-month follow-up, corresponding to an attrition rate of 45.2%. The *a priori* sample size calculation indicated that 322 participants per group (644 total) were required after allowing for up to 10% loss to follow-up. Based on this sample size requirement, the attrition rate relative to the target follow-up sample was 2.0%.

All included studies reported attrition rates of less than 20%, and the reasons for loss to follow-up were clearly described in the Consolidated Standards of Reporting Trials diagrams of the primary studies. Five studies explicitly reported using an intention-to-treat approach to handle missing data. The total number of participants allocated to the intervention and control groups was 2714, of whom 2557 were included in the analysis (Table 2).

### Intervention

All six included studies focused on providing behavioural activation for individuals who reported symptoms of mild to severe depression. The behavioural activation interventions ranged from 5 to 14 sessions, delivered weekly or bi-weekly over a period of 2 to 6 months. Session durations varied from 25 to 60 min. The mode of delivery differed across the studies, including face-to-face, telephone, video conference and online formats. Two studies delivered behavioural activation face to face with participants,^36,37^ two studies delivered it via telephone,^33,35^ and the remaining two studies used either an online^32^ or video conference^34^ format.

All comparators were considered acceptable for inclusion if they did not fall under the category of behavioural activation. The comparators used in the included studies varied and included enhanced usual care,^36^ online psychoeducation,^32,33^ friendly visit,^34^ mindfulness and befriending (Table 2).^35^

### Lay workers characteristics and training

In the six included studies, lay workers were also known as lay counsellors, lay peer persons or paraprofessionals, and received varying types of training and supervision. In one study, lay workers completed a 42 h course and 20 h of group training on behavioural activation, delivered by social workers and clinical psychologists followed by weekly supervision.^33^ In the study by Patel et al,^36^ lay peer workers were provided with a 3-week participatory workshop on the healthy activity programme followed by a 6-month internship in primary healthcare clinics.^36^ In addition, the lay peer workers received weekly group supervision and bi-monthly individual supervision. In another study, lay workers received 2 days of intensive training on internet-based behavioural activation, which included handling technical issues and clinical challenges.^32^ Lay workers also received printed materials and regular supervision.^32^ Another study reported that lay workers received six 2 h face-to-face group sessions weekly and were led by a social worker and a research assistant.^35^ The remaining two studies^34,37^ did not report the exact duration of training, but reported that lay workers received training with regular supervision (Table 1).

### Outcome measures

Outcomes were assessed across all included studies, using various depression scales. Three studies measured depression with the PHQ-9 scale as primary outcomes,^32,34,37^ whereas three studies used other scales, such as the Center for Epidemiologic Studies–Depression Scale^33^ and the BDI-II, with remission defined using PHQ-9 scores,^36^ the revised UCLA Loneliness Scale (Chinese version) and the PHQ-9 Chinese version.^35^

Four studies used PHQ-9 to determine the eligibility criteria based on the severity of depressive symptoms. In one study, participants were included if they scored >14 on the PHQ-9, indicating moderately severe to severe depression.^36^ Another study included participants if they scored ≥10 on the PHQ-9 and had a clinical diagnosis of major depressive disorder or persistent depressive disorder as per the Structured Clinical Interview for DSM-5.^32^ Similarly, Sikander et al^37^ included participants if they scored >10 on the PHQ-9; however, Choi et al^34^ included participants if they scored <10 on the PHQ-9, indicating no to mild symptoms of depression (with a baseline mean score of 7.2) (Table 2).

### Risk of bias and GRADE assessment

The six included studies were rated to have an overall high risk of bias. Across these studies, several sources of bias were identified. Patel et al^36^ demonstrated high risk of bias in domains 3, 4 and 5 because of insufficient handling of missing outcome data, lack of blinding of outcome assessors, and indications of selective reporting. Kwok et al^35^ raised some concerns regarding the randomisation process and lack of blinding of participants and people who delivered the intervention, whereas domain 5 was rated high risk because analyses did not follow a clearly prespecified plan and selective reporting was evident. Similarly, Choi et al^34^ presented some concerns about randomisation, high risk in outcome assessment owing to lack of blinding, and insufficient information to judge adherence to a prespecified analysis plan.

**Fig. 2:**
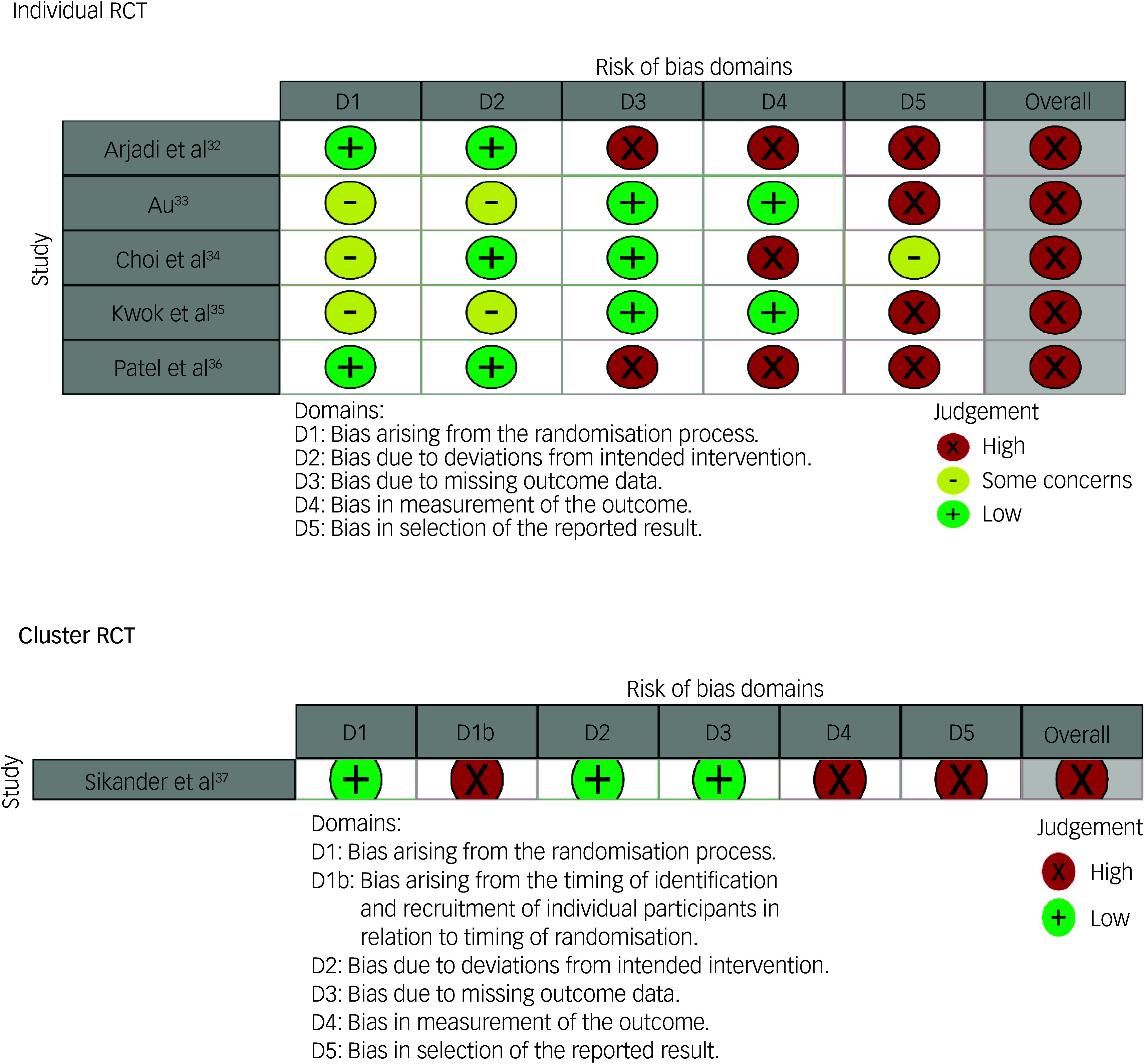
Risk of bias of the included studies. RCT, randomised controlled trial.

High risk of bias was also observed in Arjadi et al^32^ because of missing outcome data potentially related to true outcome values and unblinded outcome assessment, alongside evidence of selective reporting. Au^33^ showed some concerns in domains 1 and 2 because of limited reporting on randomisation, lack of blinding and unclear deviations from intended interventions, whereas domain 5 was judged high risk due to the absence of a prespecified analysis plan and likely selective reporting. Sikander et al^37^ was judged high risk for domain 1b because participants selection within clusters appeared to be influenced by awareness of intervention allocation, as well as high risk in domains 4 and 5 because of unblinded outcome assessment and indications of selective reporting and selective analysis (Fig. 2).

The certainty of evidence for the effect of behavioural activation delivered by lay workers to individuals diagnosed with depressive symptoms was rated as very low, downgraded because of a high risk of bias and imprecision, reflected by wide confidence intervals associated with high heterogeneity.

### Reporting of adverse events and harms

All studies reported data on adverse events during trial implementation, except one study.^33^ Two out of five studies reported no harm or adverse effects for the behavioural activation group.^34,35^ For the control group, Kwok et al^35^ reported that two participants from the mindfulness group died during the study period. It was determined that these deaths were unrelated to the trial intervention as participants had unrestricted access to their usual healthcare and were not subjected to any lifestyle restrictions.

The remaining three studies reported serious adverse events; Patel et al^36^ reported serious adverse events and prescription of antidepressant medications in both the intervention and the control group. These adverse events were similar in both the intervention and control group (*n* = 9 (4%) in the intervention group versus *n* = 10 [4%] in the control group). The reported serious adverse events included deaths, suicide attempt, and unplanned admissions to hospital from any cause. The authors noted that these events were not significantly different between groups and did not appear to be related to the intervention itself. Arjadi et al^32^ reported 15% (*n* = 47) of total participants increased suicide scores from baseline, but no serious deterioration was observed. Clinical assessments in this study found no acute suicidality, so no referrals were made, and no adverse events were reported in either group. Sikander et al^37^ reported adverse events for 43 (15%) women in the intervention group and 47 (16%) women in the control group, with at least one serious adverse event in the control group. Serious adverse events reported included death of the child of the study participant, hospital admissions (mainly involving participants’ child) and experience of physical violence; however, there was no evidence of any difference between the groups. These events did not appear to be related to the intervention itself (Table 2).

### Effectiveness of behavioural activation delivered by lay workers on reducing depression

**Fig. 3:**
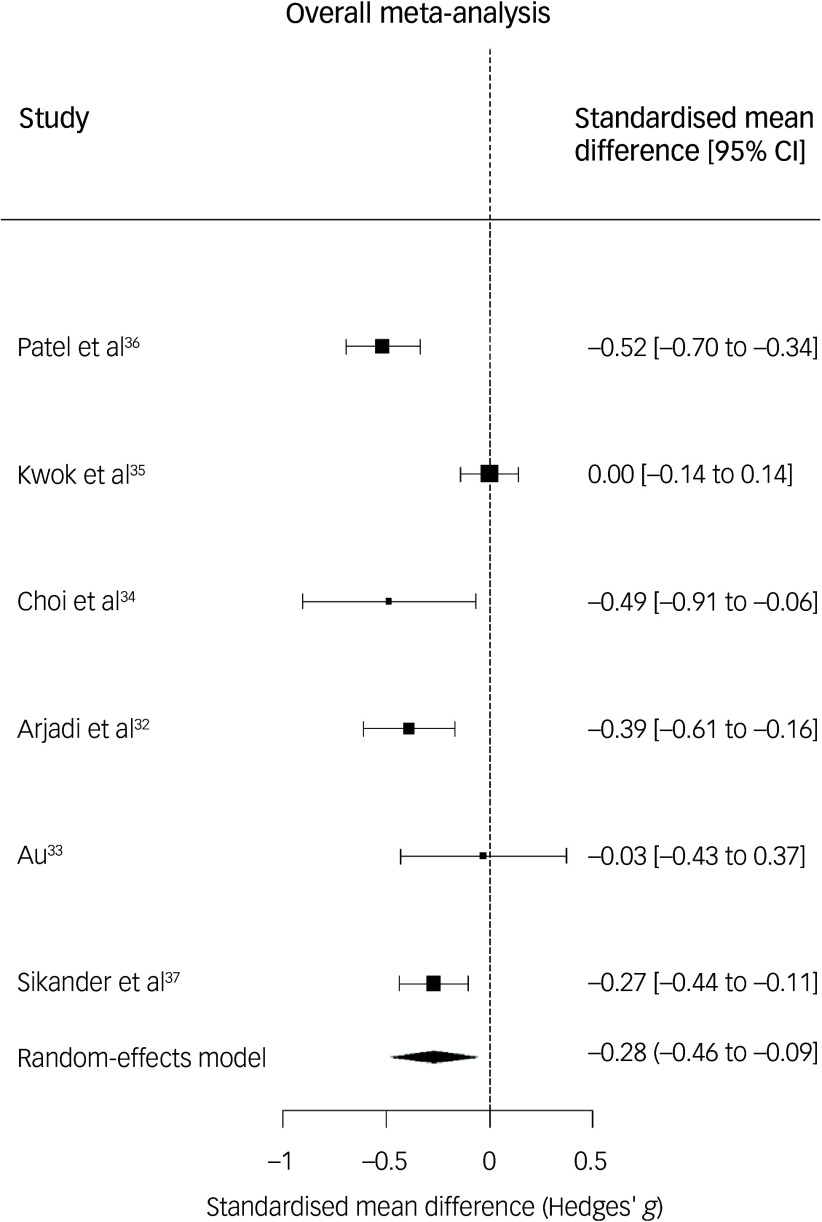
Meta-analysis forest plot.

The meta-analysis included six RCTs assessing the effectiveness of lay workers delivered behavioural activation interventions for adults diagnosed with depression. The pooled SMD was −0.28 (95% CI −0.46 to −0.09; *p* = 0.0029), indicating a small but statistically significant effect in favour for the intervention group than the control group. There was evidence of statistically significant and substantial between study heterogeneity (*I* ^2^ = 76%; Cochran’s *Q* = 24.55, d.f. = 5, *p* = 0.0002) (Fig. 3).

Findings from the meta-analysis suggest that behavioural activation may be effective in reducing depression in adults when delivered by lay workers. However, because of the high risk of bias in the included studies, these findings are based on low-quality evidence and should be interpreted with appropriate uncertainty about the effectiveness of behavioural activation delivered by lay workers for people diagnosed with depression.

### Subgroup analysis

**Fig. 4:**
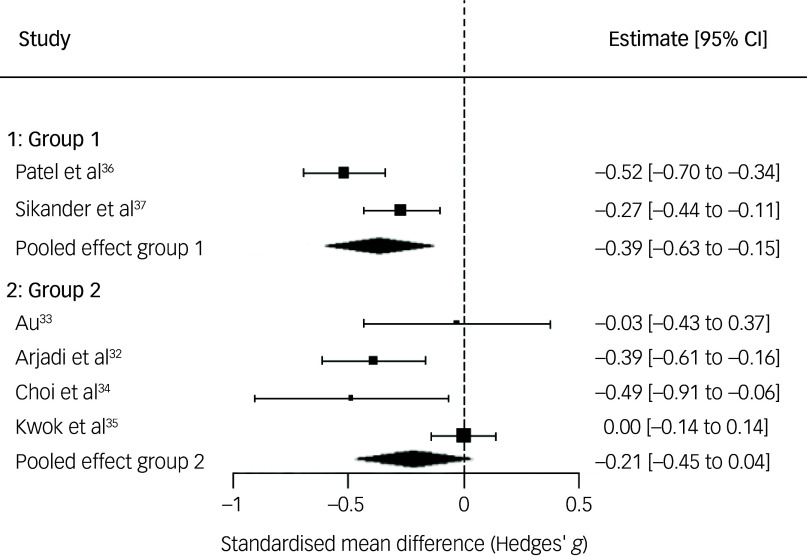
Subgroup analysis.

A subgroup analysis was conducted to explore potential sources of heterogeneity in the pooled estimate of the effect of behavioural activation on depressive symptoms, based on comparator type (enhanced usual care versus active psychoeducation). In group 1 (Patel et al^36^ and Sikander et al^37^), behavioural activation was associated with a statistically significant, moderate reduction in symptoms (*g* = −0.39). However, group 2 (the remaining four studies) demonstrated a smaller, non-significant effect (*g* = −0.21). These findings indicate that the effectiveness of behavioural activation may vary according to comparator type and study characteristics. The results of these subgroup analyses are presented in Fig. 4 and Table 3.

**Table 3:** Subgroup analysis using R Metafor

Study ID			Effect (SMD) Random effect model, 95% CI		
Group 1 (BA versus EUC)					
Patel et al^36^				−0.52 [−0.70, −0.34]	
Sikander et al^37^				−0.27 [−0.44, −0.11]	
Pooled Effect				−0.39 [−0.63, −0.15]	
Group 2 (BA versus PsycEd)					
Au^33^				0.03 [−0.43, 0.37]	
Arjadi et al^32^				−0.39 [−0.61, −0.16]	
Choi et al^34^				−0.49 [−0.91, −0.06]	
Kwok et al^35^				0.00 [−0.14, 0.14]	
Pooled Effect				−0.21 [−0.45, 0.04]	
Group 1 model results					
estimate	se	zval	pval	ci.lb	ci.ub
−0.3914	0.1235	−3.1707	0.0015	−0.6334	−0.1495 **
Group 2 model results					
estimate	se	zval	pval	ci.lb	ci.ub
−0.2057	0.1247	−1.6502	0.0989	−0.4501	0.0386

**Group 1:** Random-Effects Model (*k* = 2; tau^2 estimator: REML). tau^2 (estimated amount of total heterogeneity): 0.0228 (SE = 0.0431). tau (square root of estimated tau^2 value): 0.1509. I^2 (total heterogeneity/total variability): 74.70%. H^2 (total variability/sampling variability): 3.95. Test for Heterogeneity: Q(df = 1) = 3.9518, p-val = 0.0468.

**Group 2:** Random-Effects Model (*k* = 4; tau^2 estimator: REML). tau^2 (estimated amount of total heterogeneity): 0.0404 (SE = 0.0508). tau (square root of estimated tau^2 value): 0.2011. I^2 (total heterogeneity/total variability): 70.26%. H^2 (total variability/sampling variability): 3.36. Test for Heterogeneity: Q(df = 3) = 11.3799, *p*-val = 0.0098. Signif. codes: 0 ‘***’ 0.001 ‘**’ 0.01 ‘*’ 0.05 ‘.’ 0.1 ‘ ’ 1. SMD = Standardized Mean Difference; CI = confidence interval; *I* ^2^ = proportion of heterogeneity; Q-test evaluates within-subgroup heterogeneity.

## Discussion

The current systemic review and meta-analysis of RCTs assessed the effectiveness of behavioural activation interventions delivered by lay workers in reducing depressive symptoms in adults diagnosed with depression. Six RCTs met the inclusion criteria and were included in the meta-analysis. The pooled SMD was −0.28 (95% CI −0.46 to −0.09; *p* = 0.0029), indicating a small but statistically significant effect in favour for the intervention group than the control group. However, there was evidence of statistically significant and substantial between study heterogeneity (*I* ^2^ = 76%; Cochran’s *Q* = 24.55, d.f. = 5, *p* = 0.0002), suggests effectiveness may vary considerably across studies.

The included studies varied in various aspects, including participant demographics, behavioural activation duration and delivery, and type of comparators. Participants were randomly assigned, and the included studies reported that the attrition rate was <20% with a rational for lost to follow-up participants, which means participants were engaged with the behavioural activation intervention. Five of the included studies measured depressive symptoms as the primary outcome, with data collected and reported according to the study protocols. Although the intervention showed a small but statistically significant effect on depressive symptoms, the certainty of evidence was judged to be very low because of methodological limitations and a high risk of bias in the included studies. Therefore, these findings should be interpreted with caution and cannot be used to draw definitive conclusions about the effectiveness of behavioural activation delivered by lay workers for individuals with depression.

The behavioural activation interventions ranged from 5 to 14 sessions, delivered weekly or bi-weekly over a period of 2–6 months, with session durations varying from 25 to 60 min. Behavioural activation was effectively delivered using various modalities such as face-to-face, telephone, video conference and online formats. This flexibility in delivery format and structure highlights the adaptability of behavioural activation to diverse populations specially in low-resource and primary care settings. Although three studies reported moderate positive effects,^32,36,37^ the remaining studies showed minimal to no effect. These studies^32,36,37^ shared several characteristics, including larger sample sizes, and were conducted in low- and middle-income countries, where access to mental health services is limited and shortages of mental health professionals are common.^38,39^ In contrast, the other three studies reported minimal to no effect of behavioural activation delivered by lay workers.^33–35^ These studies were different in several aspects; all were conducted in high-income countries, behavioural activation was delivered via telephone or video conference-based modalities, and sample size varied (although the study by Kwok et al^35^ had a large sample). Differences in outcomes may reflect heterogeneity in population characteristics, intervention delivery mode, and fidelity of implementation, or context-specific effectiveness of behavioural activation, rather than indicating a uniformly effective intervention.

Overall, the evidence remains inconclusive regarding the effectiveness of behavioural activation when delivered by lay workers. Nevertheless, the pooled SMD across the included studies indicates a small but statistically significant reduction in depressive symptoms associated with lay worker-delivered behavioural activation. Importantly, despite growing evidence supporting the effectiveness of digital self-guided interventions, lay delivered behavioural activation approaches remain of interest because of lay workers’ potential to provide human support, enhance engagement in daily activities and adherence, and facilitate cultural and contextual tailoring. These features may be particularly relevant for individuals with limited digital literacy, restricted access to technology or more complex psychological needs, as well as in settings where digital infrastructure is insufficient.

Although all included studies evaluated the effectiveness of behavioural activation when delivered by lay workers, these studies varied in the extent and type of training and ongoing supervision allocated to lay workers. Effective outcomes appeared to correlate with more comprehensive training and ongoing supervision, which support the importance of adequate training preparation and ongoing support for lay workers.^32,33,36^ These studies reported effective behavioural activation with weekly ongoing supervision. However, approaches to training and supporting lay workers differed across contexts, limiting the generalisability of any single model. As a result, our findings cannot specify a particular training approach for local implementation, which requires additional context-specific co-design and assessment based on the available resources, workforce capacity and cultural considerations.

No evidence of serious adverse events/harm directly related to the behavioural activation interventions were reported. Adverse events were unrelated to the behavioural activation intervention and were distributed roughly equally across both the intervention and control groups. These findings suggest that behavioural activation, when delivered by lay workers in similar contexts, may be a safe intervention to manage depression for adults, particularly in settings with limited access to specialist mental health professionals. Overall, behavioural activation delivered by lay workers appears to be a low-risk intervention that can be delivered across diverse settings.

### Strengths and limitations

Our systematic review and meta-analysis have several strengths. The review was conducted by experienced researchers and guided by a prespecified protocol prospectively registered with PROSPERO and adhered to PRISMA statement guidelines.

A limitation and challenge across all the included studies was the inability to mask or blind participants because of the nature of the interventions. Another issue was the lack of detailed study descriptions, which led to insufficient information to verify whether any outcomes were selectively reported. The quality of the evidence in all included studies was poor because of high risk of bias. All studies were assessed as having a high risk of bias mainly arising from the measurement of outcomes and the selective reporting of results. These limitations affect the overall confidence in the review findings, and highlight the need for more rigorously designed trials in the future.

Limitations arising from the review process also need to be acknowledged. Although the search strategy was comprehensive and designed to capture all relevant study, there is a possibility that some eligible studies were missed during title and abstract screening. In cases where studies used multiple scales to measure the same outcome, only the most used scale was selected for analysis. Although no language restriction was planned, only studies with English-language abstracts were screened for inclusion. This may have introduced potential language bias, as studies without English abstracts could have been missed. This limitation should be considered when interpreting the findings.

## Future research

The findings from this review and meta-analysis suggest that behavioural activation, when delivered by trained lay workers, may offer an effective approach for reducing depressive symptoms in adults. These findings are of importance, particularly in low- and middle-income settings with limited access to specialist mental healthcare professionals. Behavioural activation is a theory driven and empirically supported intervention that is grounded in behavioural models of depression. However, because of the high risk of bias and heterogeneity of the included studies, these findings should be interpreted with caution. Across the included studies, source of risk of bias included limitations in the randomisation process, lack of blinding of outcome assessors, incomplete or inadequate handling of outcome data, and selective reporting. These methodological weaknesses may have led to an overestimation of treatment effects and reduce confidence in the pooled estimates. In addition, based on a Cochrane review reporting moderate certainty that behavioural activation produces a moderate to large effect size, and our systematic review that has found no evidence of reported harm and demonstrated strong participants engagement, we propose that a well-designed implementation RCT and rigorous training protocols to train lay workers to deliver behavioural activation are needed, especially in low-resource and primary care and community settings.

Future research should prioritise well-designed, high-quality RCTs incorporating robust randomisation procedures, strategies to minimise occurrence and appropriate handling of missing data, and clearly predefined analysis plans, to evaluate the effectiveness of behavioural activation delivered by lay workers for individuals reported depressive symptoms. Greater harmonisation in the measurement of depressive symptoms across studies would also strengthen the evidence base and facilitate comparability and synthesis, although emerging resources such as harmonisation tools may help mitigate this challenge. Given the current evidence is very weak limited by high risk of bias and substantial heterogeneity, future studies should ensure rigorous methodological standards.

## Supporting information

10.1192/bjo.2026.12018.sm001 Othman et al. supplementary material 1 Othman et al. supplementary material

10.1192/bjo.2026.12018.sm002 Othman et al. supplementary material 2 Othman et al. supplementary material

## Data Availability

As this is a systematic review, it utilised data previously reported in the primary literature or in public domain. All summary data supporting the findings are provided in the text and the Supplementary Material.

## Supplementary material

The supplementary material is available online at https://doi.org/10.1192/bjo.2026.12018
