# Supplementary material for: Effectiveness of lay workers delivering behavioural activation for people with depression: systematic review and meta-analysis
Source: BJPsych Open. 2026 Jun 25;12(4):e170. doi: 10.1192/bjo.2026.12018 (PMC13312275; doi:10.1192/bjo.2026.12018)
Supplement: Othman et al. supplementary material 1 — Othman et al. supplementary material [file S2056472426120183sup001.docx]

**Link:**
[Click to run search](https://aus01.safelinks.protection.outlook.com/?url=https%3A%2F%2Fovidsp.ovid.com%2Fovidweb.cgi%3FT%3DJS%26NEWS%3DN%26PAGE%3Dmain%26SHAREDSEARCHID%3D3t07egwXeMRmCOEq5EZYc7dSUbQJ3pLlyK4bjuD878xgwUNLo1kjDiaCSaUPuV9Hn&data=05%7C02%7Cs.othman%40ecu.edu.au%7Ca66f3633a5c94a48e83a08dd18c29a82%7C9bcb323d7fa345e7a36f6d9cfdbcc272%7C1%7C0%7C638693946297634878%7CUnknown%7CTWFpbGZsb3d8eyJFbXB0eU1hcGkiOnRydWUsIlYiOiIwLjAuMDAwMCIsIlAiOiJXaW4zMiIsIkFOIjoiTWFpbCIsIldUIjoyfQ%3D%3D%7C0%7C%7C%7C&sdata=0UFtTcghc0%2F3kDhIljo52o9jO%2Bzv50Z2AwOyi4yOSPU%3D&reserved=0)
The above Jumpstart will only work for users who have access to this specific database.


**Database:**
Ovid MEDLINE(R) ALL <1946 to December 06, 2024>

| **#** | **Query** | **Results from 10 Dec 2024** |
| --- | --- | --- |
| 1 | exp Community Health Workers/ | 7,037 |
| 2 | exp Home Health Aides/ or exp Homemaker Services/ | 1,257 |
| 3 | exp Volunteers/ | 40,096 |
| 4 | exp Home Nursing/ | 9,567 |
| 5 | *Health Personnel/ | 37,616 |
| 6 | exp Peer Group/ | 26,047 |
| 7 | exp Social Support/ | 83,584 |
| 8 | ((lay or voluntary or volunteer* or untrained or unlicensed or nonprofessional* or non professional*) adj5 (worker* or visitor* or attendant* or aide or aides or support* or person* or helper* or carer* or caregiver* or care giver* or consultant* or assistant* or visit*)).ti,ab,kf. | 11,076 |
| 9 | (lay health worker* or lay volunteer*).ti,ab,kf. | 679 |
| 10 | (trained adj3 (volunteer* or health worker*)).ti,ab,kf. | 2,365 |
| 11 | ((community or village*) adj3 (health worker* or health care worker* or healthcare worker*)).ti,ab,kf. | 9,513 |
| 12 | (community adj3 (volunteer* or aide or aides or support)).ti,ab,kf. | 12,248 |
| 13 | (peer adj (volunteer* or counsel* or support or intervention*)).ti,ab,kf. | 9,699 |
| 14 | (church based adj3 (intervention* or program* or counsel*)).ti,ab,kf. | 217 |
| 15 | (linkworker* or link worker*).ti,ab,kf. | 195 |
| 16 | barefoot doctor*.ti,ab,kf. | 148 |
| 17 | (home adj (care or aide or aides or nursing or support or intervention* or treatmen* or visit*)).ti,ab,kf. | 38,963 |
| 18 | ((care or aide or aides or nursing or support or intervention* or treatment* or visit*) adj3 (lay or volunteer* or voluntary)).ti,ab,kf. | 6,431 |
| 19 | (community worker* or community health* worker* or community health care worker* or community volunteer* or non specialist* or nonspecialist*).ti,ab,kf. | 13,432 |
| 20 | (community based worker* or community based health* worker* or community based health care worker* or community based volunteer*).ti,ab,kf. | 331 |
| 21 | (lay adj3 (counsellor* or counsellor* or counseling or counselling or coach* or intervention* or support or outreach or delivered or staff or led or provider* or based or volunteer* or mentor* or educator* or visitor* or adviser* or advisor* or facilitator* or person*)).ti,ab,kf. | 3,083 |
| 22 | ((lay adj worker*) or (lay adj health* worker*) or (lay adj health care worker*)).ti,ab,kf. | 721 |
| 23 | (village based worker* or village based health* worker* or village based health care worker*).ti,ab,kf. | 20 |
| 24 | ((peer adj worker*) or (peer adj health* worker*) or (peer adj health care worker*)).ti,ab,kf. | 295 |
| 25 | (peer adj (counselor* or counsellor* or counseling or counselling or coach* or intervention* or support or outreach or delivered or staff or led or provider* or based or volunteer* or mentor* or educator* or visitor* or adviser* or advisor* or facilitator* or personnel)).ti,ab,kf. | 14,394 |
| 26 | (volunteer* adj3 (counselor* or counsellor* or counseling or counselling or coach* or intervention* or support or outreach or delivered or staff or led or provider* or based or mentor* or educator* or visitor* or adviser* or advisor*)).ti,ab,kf. | 4,385 |
| 27 | ((non professional* or nonprofessional* or paraprofessional*) adj3 (counselor* or counsellor* or counseling or counselling or coach* or intervention* or support or outreach or delivered or staff or led or provider* or based or volunteer* or mentor* or educator* or visitor* or adviser* or advisor* or facilitator* or personnel)).ti,ab,kf. | 751 |
| 28 | (village worker* or village health* worker* or village health care worker*).ti,ab,kf. | 462 |
| 29 | ((outreach or support or family) adj worker*).ti,ab,kf. | 2,208 |
| 30 | ((home visit* or household visit*) adj3 (intervention* or program* or condition or non professional* or nonprofessional* or paraprofessional* or volunteer*)).ti,ab,kf. | 2,097 |
| 31 | 1 or 2 or 3 or 4 or 5 or 6 or 7 or 8 or 9 or 10 or 11 or 12 or 13 or 14 or 15 or 16 or 17 or 18 or 19 or 20 or 21 or 22 or 23 or 24 or 25 or 26 or 27 or 28 or 29 or 30 | 275,811 |
| 32 | Depression/ | 164,464 |
| 33 | Mood Disorders/ | 16,440 |
| 34 | Depressive Disorder/ | 75,684 |
| 35 | Depressive Disorder, Major/ | 41,639 |
| 36 | Depressive Disorder, Treatment-Resistant/ | 2,487 |
| 37 | Dysthymic Disorder/ | 1,186 |
| 38 | Cyclothymic Disorder/ | 801 |
| 39 | (depress* or dysthymi* or cyclothymi* or low mood or mood disorder* or affective disorder*).ti,ab,kf. | 637,596 |
| 40 | 32 or 33 or 34 or 35 or 36 or 37 or 38 or 39 | 683,006 |
| 41 | 31 and 40 | 21,826 |
| 42 | exp Behavior Therapy/ | 95,193 |
| 43 | behavio* activat*.ti,ab,kf. | 2,944 |
| 44 | (behavio* activat* or BATD).ti,ab,kf. | 2,951 |
| 45 | (behavio* adj3 (reinforce* or re-inforce*)).ti,ab,kf. | 3,820 |
| 46 | (behavio* adj2 (contracting or modif*)).ti,ab,kf. | 12,324 |
| 47 | reinforc*.ti,ab,kf. | 155,214 |
| 48 | ((positive adj1 reinforc*) or (reinforc* adj3 (environment* or experience*))).ti,ab,kf. | 3,966 |
| 49 | (activit* adj2 schedul*).ti,ab,kf. | 760 |
| 50 | ((pleas* or enjoyable or rewarding) adj3 (activit* or event?)).ti,ab,kf. | 1,664 |
| 51 | ((operant or instrumental) adj (conditioning or learning)).ti,ab,kf. | 3,970 |
| 52 | (positive interaction* or avoida* coping or environmental contingenc* or contingency management).ti,ab,kf. | 6,895 |
| 53 | functional analysis.ti,ab,kf. | 33,459 |
| 54 | behavio*.mp. and (self adj (evaluat* or monitor*)).ti,ab,kf. | 5,375 |
| 55 | (behavio* adj (counsel* or intervention* or train* or treatment* or therap* or psychotherap*)).ti,ab,kf. | 55,773 |
| 56 | (mood adj3 monitor*).ti,ab,kf. | 379 |
| 57 | 42 or 43 or 44 or 45 or 46 or 47 or 48 or 49 or 50 or 51 or 52 or 53 or 54 or 55 or 56 | 334,708 |
| 58 | 41 and 57 | **1,475** |

**Link:**
[Click to run search](https://aus01.safelinks.protection.outlook.com/?url=https%3A%2F%2Fovidsp.ovid.com%2Fovidweb.cgi%3FT%3DJS%26NEWS%3DN%26PAGE%3Dmain%26SHAREDSEARCHID%3D329mbK0xvOB4tHEnmDB3LgPIixqPWrQ8PUkAVMrIdnsZwbWYvpNBAqFreo8nHYGxl&data=05%7C02%7Cs.othman%40ecu.edu.au%7Cb78ae662d42d4d1a85c408dd18d03685%7C9bcb323d7fa345e7a36f6d9cfdbcc272%7C1%7C0%7C638694004751763466%7CUnknown%7CTWFpbGZsb3d8eyJFbXB0eU1hcGkiOnRydWUsIlYiOiIwLjAuMDAwMCIsIlAiOiJXaW4zMiIsIkFOIjoiTWFpbCIsIldUIjoyfQ%3D%3D%7C0%7C%7C%7C&sdata=fDSba5nQS5goX3WLNKx71TpQTGVVHYxTuauHoZ21oog%3D&reserved=0)
The above Jumpstart will only work for users who have access to this specific database.


**Database:**
Embase <1974 to 2024 December 05>

| **#** | **Query** | **Results from 10 Dec 2024** |
| --- | --- | --- |
| 1 | exp health auxiliary/ | 10,778 |
| 2 | health care personnel/ or *home care/ | 306,307 |
| 3 | volunteer/ | 61,577 |
| 4 | peer group/ | 33,286 |
| 5 | *social support/ | 28,370 |
| 6 | ((lay or voluntary or volunteer* or untrained or unlicensed or nonprofessional* or non professional*) adj5 (worker* or visitor* or attendant* or aide or aides or support* or person* or helper* or carer* or caregiver* or care giver* or consultant* or assistant* or visit*)).ti,ab,kf. | 14,448 |
| 7 | (lay health worker* or lay volunteer*).ti,ab,kf. | 809 |
| 8 | (trained adj3 (volunteer* or health worker*)).ti,ab,kf. | 3,211 |
| 9 | ((community or village*) adj3 (health worker* or health care worker* or healthcare worker*)).ti,ab,kf. | 11,255 |
| 10 | (community adj3 (volunteer* or aide or aides or support)).ti,ab,kf. | 15,823 |
| 11 | (peer adj (volunteer* or counsel* or support or intervention*)).ti,ab,kf. | 12,843 |
| 12 | (church based adj3 (intervention* or program* or counsel*)).ti,ab,kf. | 248 |
| 13 | (linkworker* or link worker*).ti,ab,kf. | 251 |
| 14 | barefoot doctor*.ti,ab,kf. | 138 |
| 15 | (home adj (care or aide or aides or nursing or support or intervention* or treatmen* or visit*)).ti,ab,kf. | 48,699 |
| 16 | ((care or aide or aides or nursing or support or intervention* or treatment* or visit*) adj3 (lay or volunteer* or voluntary)).ti,ab,kf. | 9,475 |
| 17 | (community worker* or community health* worker* or community health care worker* or community volunteer* or non specialist* or nonspecialist*).ti,ab,kf. | 16,393 |
| 18 | (community based worker* or community based health* worker* or community based health care worker* or community based volunteer*).ti,ab,kf. | 385 |
| 19 | (lay adj3 (counsellor* or counsellor* or counseling or counselling or coach* or intervention* or support or outreach or delivered or staff or led or provider* or based or volunteer* or mentor* or educator* or visitor* or adviser* or advisor* or facilitator* or person*)).ti,ab,kf. | 4,028 |
| 20 | ((lay adj worker*) or (lay adj health* worker*) or (lay adj health care worker*)).ti,ab,kf. | 809 |
| 21 | (village based worker* or village based health* worker* or village based health care worker*).ti,ab,kf. | 21 |
| 22 | ((peer adj worker*) or (peer adj health* worker*) or (peer adj health care worker*)).ti,ab,kf. | 347 |
| 23 | (peer adj (counselor* or counsellor* or counseling or counselling or coach* or intervention* or support or outreach or delivered or staff or led or provider* or based or volunteer* or mentor* or educator* or visitor* or adviser* or advisor* or facilitator* or personnel)).ti,ab,kf. | 18,912 |
| 24 | (volunteer* adj3 (counselor* or counsellor* or counseling or counselling or coach* or intervention* or support or outreach or delivered or staff or led or provider* or based or mentor* or educator* or visitor* or adviser* or advisor*)).ti,ab,kf. | 6,945 |
| 25 | ((non professional* or nonprofessional* or paraprofessional*) adj3 (counselor* or counsellor* or counseling or counselling or coach* or intervention* or support or outreach or delivered or staff or led or provider* or based or volunteer* or mentor* or educator* or visitor* or adviser* or advisor* or facilitator* or personnel)).ti,ab,kf. | 837 |
| 26 | (village worker* or village health* worker* or village health care worker*).ti,ab,kf. | 467 |
| 27 | ((outreach or support or family) adj worker*).ti,ab,kf. | 2,929 |
| 28 | ((home visit* or household visit*) adj3 (intervention* or program* or condition or non professional* or nonprofessional* or paraprofessional* or volunteer*)).ti,ab,kf. | 2,394 |
| 29 | 1 or 2 or 3 or 4 or 5 or 6 or 7 or 8 or 9 or 10 or 11 or 12 or 13 or 14 or 15 or 16 or 17 or 18 or 19 or 20 or 21 or 22 or 23 or 24 or 25 or 26 or 27 or 28 | 512,518 |
| 30 | exp depression/ | 686,149 |
| 31 | exp mood disorder/ | 738,058 |
| 32 | major depression/ | 90,044 |
| 33 | treatment resistant depression/ | 6,182 |
| 34 | dysthymia/ | 9,609 |
| 35 | cyclothymia/ | 1,141 |
| 36 | (depress* or dysthymi* or cyclothymi* or low mood or mood disorder* or affective disorder*).ti,ab,kf. | 856,965 |
| 37 | 30 or 31 or 32 or 33 or 34 or 35 or 36 | 1,097,853 |
| 38 | 29 and 37 | 32,391 |
| 39 | exp behavior therapy/ | 83,796 |
| 40 | behavio* activat*.ti,ab,kf. | 3,579 |
| 41 | (behavio* activat* or BATD).ti,ab,kf. | 3,587 |
| 42 | (behavio* adj3 (reinforce* or re-inforce*)).ti,ab,kf. | 4,154 |
| 43 | (behavio* adj2 (contracting or modif*)).ti,ab,kf. | 15,791 |
| 44 | reinforc*.ti,ab,kf. | 177,186 |
| 45 | ((positive adj1 reinforc*) or (reinforc* adj3 (environment* or experience*))).ti,ab,kf. | 4,954 |
| 46 | (activit* adj2 schedul*).ti,ab,kf. | 1,082 |
| 47 | ((pleas* or enjoyable or rewarding) adj3 (activit* or event?)).ti,ab,kf. | 2,273 |
| 48 | ((operant or instrumental) adj (conditioning or learning)).ti,ab,kf. | 4,777 |
| 49 | (positive interaction* or avoida* coping or environmental contingenc* or contingency management).ti,ab,kf. | 8,332 |
| 50 | functional analysis.ti,ab,kf. | 40,473 |
| 51 | behavio*.mp. and (self adj (evaluat* or monitor*)).ti,ab,kf. | 6,952 |
| 52 | (behavio* adj (counsel* or intervention* or train* or treatment* or therap* or psychotherap*)).ti,ab,kf. | 76,363 |
| 53 | (mood adj3 monitor*).ti,ab,kf. | 541 |
| 54 | 39 or 40 or 41 or 42 or 43 or 44 or 45 or 46 or 47 or 48 or 49 or 50 or 51 or 52 or 53 | 371,269 |
| 55 | 38 and 54 | **2,121** |

**Link:**
[Click to run search](https://aus01.safelinks.protection.outlook.com/?url=https%3A%2F%2Fovidsp.ovid.com%2Fovidweb.cgi%3FT%3DJS%26NEWS%3DN%26PAGE%3Dmain%26SHAREDSEARCHID%3D6n041uTWUKaSM7hZoWfz9kJgW5GOpBIRo1at4EOoUSJLPlQrwaskdmqbcNRSyyaMt&data=05%7C02%7Cs.othman%40ecu.edu.au%7Cae6769d2f79a4147032708dd18d40b32%7C9bcb323d7fa345e7a36f6d9cfdbcc272%7C1%7C0%7C638694021204384740%7CUnknown%7CTWFpbGZsb3d8eyJFbXB0eU1hcGkiOnRydWUsIlYiOiIwLjAuMDAwMCIsIlAiOiJXaW4zMiIsIkFOIjoiTWFpbCIsIldUIjoyfQ%3D%3D%7C0%7C%7C%7C&sdata=Xpq1hs6zI4bhw0m9j0ne8giXk89iKphfGjxkhpMF9eA%3D&reserved=0)
The above Jumpstart will only work for users who have access to this specific database.


**Database:**
Ovid Emcare <1995 to 2024 Week 48>

| **#** | **Query** | **Results from 10 Dec 2024** |
| --- | --- | --- |
| 1 | exp health auxiliary/ | 5,786 |
| 2 | *home care/ | 14,933 |
| 3 | *health care personnel/ | 24,369 |
| 4 | volunteer/ | 32,647 |
| 5 | peer group/ | 16,998 |
| 6 | social support/ | 71,881 |
| 7 | ((lay or voluntary or volunteer* or untrained or unlicensed or nonprofessional* or non professional*) adj5 (worker* or visitor* or attendant* or aide or aides or support* or person* or helper* or carer* or caregiver* or care giver* or consultant* or assistant* or visit*)).ti,ab,kf. | 6,871 |
| 8 | (lay health worker* or lay volunteer*).ti,ab,kf. | 459 |
| 9 | (trained adj3 (volunteer* or health worker*)).ti,ab,kf. | 1,305 |
| 10 | ((community or village*) adj3 (health worker* or health care worker* or healthcare worker*)).ti,ab,kf. | 6,157 |
| 11 | (community adj3 (volunteer* or aide or aides or support)).ti,ab,kf. | 8,871 |
| 12 | (peer adj (volunteer* or counsel* or support or intervention*)).ti,ab,kf. | 7,979 |
| 13 | (church based adj3 (intervention* or program* or counsel*)).ti,ab,kf. | 158 |
| 14 | (linkworker* or link worker*).ti,ab,kf. | 149 |
| 15 | barefoot doctor*.ti,ab,kf. | 20 |
| 16 | (home adj (care or aide or aides or nursing or support or intervention* or treatmen* or visit*)).ti,ab,kf. | 27,184 |
| 17 | ((care or aide or aides or nursing or support or intervention* or treatment* or visit*) adj3 (lay or volunteer* or voluntary)).ti,ab,kf. | 4,312 |
| 18 | (community worker* or community health* worker* or community health care worker* or community volunteer* or non specialist* or nonspecialist*).ti,ab,kf. | 7,927 |
| 19 | (community based worker* or community based health* worker* or community based health care worker* or community based volunteer*).ti,ab,kf. | 212 |
| 20 | (lay adj3 (counsellor* or counsellor* or counseling or counselling or coach* or intervention* or support or outreach or delivered or staff or led or provider* or based or volunteer* or mentor* or educator* or visitor* or adviser* or advisor* or facilitator* or person*)).ti,ab,kf. | 2,111 |
| 21 | ((lay adj worker*) or (lay adj health* worker*) or (lay adj health care worker*)).ti,ab,kf. | 483 |
| 22 | (village based worker* or village based health* worker* or village based health care worker*).ti,ab,kf. | 5 |
| 23 | ((peer adj worker*) or (peer adj health* worker*) or (peer adj health care worker*)).ti,ab,kf. | 259 |
| 24 | (peer adj (counselor* or counsellor* or counseling or counselling or coach* or intervention* or support or outreach or delivered or staff or led or provider* or based or volunteer* or mentor* or educator* or visitor* or adviser* or advisor* or facilitator* or personnel)).ti,ab,kf. | 11,591 |
| 25 | (volunteer* adj3 (counselor* or counsellor* or counseling or counselling or coach* or intervention* or support or outreach or delivered or staff or led or provider* or based or mentor* or educator* or visitor* or adviser* or advisor*)).ti,ab,kf. | 3,233 |
| 26 | ((non professional* or nonprofessional* or paraprofessional*) adj3 (counselor* or counsellor* or counseling or counselling or coach* or intervention* or support or outreach or delivered or staff or led or provider* or based or volunteer* or mentor* or educator* or visitor* or adviser* or advisor* or facilitator* or personnel)).ti,ab,kf. | 532 |
| 27 | (village worker* or village health* worker* or village health care worker*).ti,ab,kf. | 197 |
| 28 | ((outreach or support or family) adj worker*).ti,ab,kf. | 1,980 |
| 29 | ((home visit* or household visit*) adj3 (intervention* or program* or condition or non professional* or nonprofessional* or paraprofessional* or volunteer*)).ti,ab,kf. | 1,750 |
| 30 | 1 or 2 or 3 or 4 or 5 or 6 or 7 or 8 or 9 or 10 or 11 or 12 or 13 or 14 or 15 or 16 or 17 or 18 or 19 or 20 or 21 or 22 or 23 or 24 or 25 or 26 or 27 or 28 or 29 | 201,106 |
| 31 | exp depression/ | 228,579 |
| 32 | exp mood disorder/ | 245,054 |
| 33 | exp major depression/ | 31,561 |
| 34 | treatment resistant depression/ | 1,455 |
| 35 | dysthymia/ | 3,718 |
| 36 | cyclothymia/ | 439 |
| 37 | (depress* or dysthymi* or cyclothymi* or low mood or mood disorder* or affective disorder*).ti,ab,kf. | 260,225 |
| 38 | 31 or 32 or 33 or 34 or 35 or 36 or 37 | 339,497 |
| 39 | 30 and 38 | 23,882 |
| 40 | exp behavior therapy/ | 36,309 |
| 41 | behavio* activat*.ti,ab,kf. | 1,486 |
| 42 | (behavio* activat* or BATD).ti,ab,kf. | 1,489 |
| 43 | (behavio* adj3 (reinforce* or re-inforce*)).ti,ab,kf. | 1,408 |
| 44 | (behavio* adj2 (contracting or modif*)).ti,ab,kf. | 5,024 |
| 45 | reinforc*.ti,ab,kf. | 49,372 |
| 46 | ((positive adj1 reinforc*) or (reinforc* adj3 (environment* or experience*))).ti,ab,kf. | 1,713 |
| 47 | (activit* adj2 schedul*).ti,ab,kf. | 410 |
| 48 | ((pleas* or enjoyable or rewarding) adj3 (activit* or event?)).ti,ab,kf. | 1,040 |
| 49 | ((operant or instrumental) adj (conditioning or learning)).ti,ab,kf. | 816 |
| 50 | (positive interaction* or avoida* coping or environmental contingenc* or contingency management).ti,ab,kf. | 4,016 |
| 51 | functional analysis.ti,ab,kf. | 5,431 |
| 52 | behavio*.mp. and (self adj (evaluat* or monitor*)).ti,ab,kf. | 3,595 |
| 53 | (behavio* adj (counsel* or intervention* or train* or treatment* or therap* or psychotherap*)).ti,ab,kf. | 36,546 |
| 54 | (mood adj3 monitor*).ti,ab,kf. | 179 |
| 55 | 40 or 41 or 42 or 43 or 44 or 45 or 46 or 47 or 48 or 49 or 50 or 51 or 52 or 53 or 54 | 121,605 |
| 56 | 39 and 55 | **1,626** |

**Link:**
[Click to run search](https://aus01.safelinks.protection.outlook.com/?url=https%3A%2F%2Fovidsp.ovid.com%2Fovidweb.cgi%3FT%3DJS%26NEWS%3DN%26PAGE%3Dmain%26SHAREDSEARCHID%3D4XDZUnMvFDTLveLt7Ab1hQhLpRSCyD6SHCIFczwOtgLHlBT3ugkLIFjHGPIRExf65&data=05%7C02%7Cs.othman%40ecu.edu.au%7C4e15e6a15331451d5b1f08dd18ed6222%7C9bcb323d7fa345e7a36f6d9cfdbcc272%7C1%7C0%7C638694130045432331%7CUnknown%7CTWFpbGZsb3d8eyJFbXB0eU1hcGkiOnRydWUsIlYiOiIwLjAuMDAwMCIsIlAiOiJXaW4zMiIsIkFOIjoiTWFpbCIsIldUIjoyfQ%3D%3D%7C0%7C%7C%7C&sdata=cD5wulAY1e7Fu9iYQFFnIpSoVewk%2F17NcUGDg8QOeYU%3D&reserved=0)
The above Jumpstart will only work for users who have access to this specific database.


**Database:**
APA PsycInfo <1806 to December 2024 Week 1>

| **#** | **Query** | **Results from 10 Dec 2024** |
| --- | --- | --- |
| 1 | exp Home Care Personnel/ | 604 |
| 2 | exp Caregivers/ | 40,898 |
| 3 | exp Volunteers/ | 6,469 |
| 4 | exp Health Personnel/ | 286,543 |
| 5 | exp Peers/ | 19,047 |
| 6 | exp Social Support/ | 47,806 |
| 7 | exp Nonprofessional Personnel/ or exp Paraprofessional Personnel/ | 3,137 |
| 8 | ((lay or voluntary or volunteer* or untrained or unlicensed or nonprofessional* or non professional*) adj5 (worker* or visitor* or attendant* or aide or aides or support* or person* or helper* or carer* or caregiver* or care giver* or consultant* or assistant* or visit*)).ti,ab. | 6,766 |
| 9 | (lay health worker* or lay volunteer*).ti,ab. | 245 |
| 10 | (trained adj3 (volunteer* or health worker*)).ti,ab. | 628 |
| 11 | ((community or village*) adj3 (health worker* or health care worker* or healthcare worker*)).ti,ab. | 1,903 |
| 12 | (community adj3 (volunteer* or aide or aides or support)).ti,ab. | 9,004 |
| 13 | (peer adj (volunteer* or counsel* or support or intervention*)).ti,ab. | 7,557 |
| 14 | (church based adj3 (intervention* or program* or counsel*)).ti,ab. | 196 |
| 15 | (linkworker* or link worker*).ti,ab. | 68 |
| 16 | barefoot doctor*.ti,ab. | 10 |
| 17 | (home adj (care or aide or aides or nursing or support or intervention* or treatmen* or visit*)).ti,ab. | 13,448 |
| 18 | ((care or aide or aides or nursing or support or intervention* or treatment* or visit*) adj3 (lay or volunteer* or voluntary)).ti,ab. | 3,126 |
| 19 | (community worker* or community health* worker* or community health care worker* or community volunteer* or non specialist* or nonspecialist*).ti,ab. | 3,991 |
| 20 | (community based worker* or community based health* worker* or community based health care worker* or community based volunteer*).ti,ab. | 104 |
| 21 | (lay adj3 (counsellor* or counsellor* or counseling or counselling or coach* or intervention* or support or outreach or delivered or staff or led or provider* or based or volunteer* or mentor* or educator* or visitor* or adviser* or advisor* or facilitator* or person*)).ti,ab. | 1,991 |
| 22 | ((lay adj worker*) or (lay adj health* worker*) or (lay adj health care worker*)).ti,ab. | 247 |
| 23 | (village based worker* or village based health* worker* or village based health care worker*).ti,ab. | 5 |
| 24 | ((peer adj worker*) or (peer adj health* worker*) or (peer adj health care worker*)).ti,ab. | 242 |
| 25 | (peer adj (counselor* or counsellor* or counseling or counselling or coach* or intervention* or support or outreach or delivered or staff or led or provider* or based or volunteer* or mentor* or educator* or visitor* or adviser* or advisor* or facilitator* or personnel)).ti,ab. | 11,413 |
| 26 | (volunteer* adj3 (counselor* or counsellor* or counseling or counselling or coach* or intervention* or support or outreach or delivered or staff or led or provider* or based or mentor* or educator* or visitor* or adviser* or advisor*)).ti,ab. | 2,762 |
| 27 | ((non professional* or nonprofessional* or paraprofessional*) adj3 (counselor* or counsellor* or counseling or counselling or coach* or intervention* or support or outreach or delivered or staff or led or provider* or based or volunteer* or mentor* or educator* or visitor* or adviser* or advisor* or facilitator* or personnel)).ti,ab. | 1,122 |
| 28 | (village worker* or village health* worker* or village health care worker*).ti,ab. | 38 |
| 29 | ((outreach or support or family) adj worker*).ti,ab. | 1,611 |
| 30 | ((home visit* or household visit*) adj3 (intervention* or program* or condition or non professional* or nonprofessional* or paraprofessional* or volunteer*)).ti,ab. | 1,541 |
| 31 | 1 or 2 or 3 or 4 or 5 or 6 or 7 or 8 or 9 or 10 or 11 or 12 or 13 or 14 or 15 or 16 or 17 or 18 or 19 or 20 or 21 or 22 or 23 or 24 or 25 or 26 or 27 or 28 or 29 or 30 | 388,108 |
| 32 | exp "Depression (Emotion)"/ or exp Major Depression/ or exp Treatment Resistant Depression/ | 202,320 |
| 33 | exp Affective Disorders/ | 190,216 |
| 34 | exp Dysthymic Disorder/ | 1,545 |
| 35 | exp Cyclothymic Disorder/ | 232 |
| 36 | (depress* or dysthymi* or cyclothymi* or low mood or mood disorder* or affective disorder*).ti,ab. | 395,041 |
| 37 | 32 or 33 or 34 or 35 or 36 | 406,145 |
| 38 | 31 and 37 | 27,998 |
| 39 | Behavior Therapy/ or Behavioral Activation System/ | 17,465 |
| 40 | (behavio* activat* or BATD).ti,ab. | 2,858 |
| 41 | (behavio* adj3 (reinforce* or re-inforce*)).ti,ab. | 5,336 |
| 42 | (behavio* adj2 (contracting or modif*)).ti,ab. | 9,848 |
| 43 | behavio* activat*.ti,ab. | 2,857 |
| 44 | reinforc*.ti,ab. | 90,048 |
| 45 | ((positive adj1 reinforc*) or (reinforc* adj3 (environment* or experience*))).ti,ab. | 5,140 |
| 46 | (activit* adj2 schedul*).ti,ab. | 688 |
| 47 | ((pleas* or enjoyable or rewarding) adj3 (activit* or event?)).ti,ab. | 2,106 |
| 48 | ((operant or instrumental) adj (conditioning or learning)).ti,ab. | 5,508 |
| 49 | (positive interaction* or avoida* coping or environmental contingenc* or contingency management).ti,ab. | 6,941 |
| 50 | functional analysis.ti,ab. | 3,393 |
| 51 | behavio*.mp. and (self adj (evaluat* or monitor*)).ti,ab. | 6,951 |
| 52 | (behavio* adj (counsel* or intervention* or train* or treatment* or therap* or psychotherap*)).ti,ab. | 62,993 |
| 53 | (mood adj3 monitor*).ti,ab. | 327 |
| 54 | 39 or 40 or 41 or 42 or 43 or 44 or 45 or 46 or 47 or 48 or 49 or 50 or 51 or 52 or 53 | 185,772 |
| 55 | 38 and 54 | **1,578** |

**CINAHL Ultimate (11-12-2024)**

| **Search Terms** | | **Results** |
| --- | --- | --- |
| S41 | S33 AND S40 | **1,639** |
| S40 | S34 OR S35 OR S36 OR S37 OR S38 OR S39 | 78,508 |
| S39 | (TI(self W0 (evaluat* or monitor*)) OR AB(self W0 (evaluat* or monitor*))) AND behavio* | 2,354 |
| S38 | TI ( ((“functional analysis” OR (behavio* W0 (counsel* or intervention* or train* or treatment* or therap* or psychotherap*)) OR (mood N2 monitor*)) ) OR AB ( ((“functional analysis” OR (behavio* W0 (counsel* or intervention* or train* or treatment* or therap* or psychotherap*)) OR (mood N2 monitor*)) ) | 27,452 |
| S37 | TI ( (((operant or instrumental) W0 (conditioning or learning)) OR (“positive interaction*” or avoida* “coping or environmental contingenc*” or “contingency management”)) ) OR AB ( (((operant or instrumental) W0 (conditioning or learning)) OR (“positive interaction*” or avoida* “coping or environmental contingenc*” or “contingency management”)) ) | 1,864 |
| S36 | TI ( (((positive N0 reinforc*) or (reinforc* N2 (environment* or experience*)) OR (behavio* N2 motivat*) or “motivational interviewing” OR (activit* N1 schedul*) OR ((pleas* or enjoyable or rewarding) N2 (activit* or event?))) ) OR AB ( (((positive N0 reinforc*) or (reinforc* N2 (environment* or experience*)) OR (behavio* N2 motivat*) or “motivational interviewing” OR (activit* N1 schedul*) OR ((pleas* or enjoyable or rewarding) N2 (activit* or event?))) ) | 9,449 |
| S35 | TI ( (("behavio* activat*") OR ("behavio* activat*" or BATD) OR (behavio* N2 (reinforce* or "re inforce*" OR reinforce)) OR (behavio* N1 (contracting or modif*)) OR reinforc*) ) OR AB ( (("behavio* activat*") OR ("behavio* activat*" or BATD) OR (behavio* N2 (reinforce* or "re inforce*" OR reinforce)) OR (behavio* N1 (contracting or modif*)) OR reinforc*) ) | 32,986 |
| S34 | (MH (“Behavior Therapy”) | 14,210 |
| S33 | S31 AND S32 | 30,405 |
| S32 | ( (MH (Depression OR “Affective Disorders” OR “Dysthymic Disorder” OR “Cyclothymic Disorder”) ) OR TI ( (depress* or dysthymi* or cyclothymi* or “low mood” or “mood disorder*” or “affective disorder*”) ) OR AB ( (depress* or dysthymi* or cyclothymi* or “low mood” or “mood disorder*” or “affective disorder*”) ) | 236,080 |
| S31 | S1 OR S2 OR S3 OR S4 OR S5 OR S6 OR S7 OR S8 OR S9 OR S10 OR S11 OR S12 OR S13 OR S14 OR S15 OR S16 OR S17 OR S18 OR S19 OR S20 OR S21 OR S22 OR S23 OR S24 OR S25 OR S26 OR S27 OR S28 OR S29 OR S30 | 429,317 |
| S30 | TI ( (("home visit*" or "household visit*") N2 (intervention* or program* or condition or "non professional*" or nonprofessional* or paraprofessional* or volunteer*)) ) OR AB ( (("home visit*" or "household visit*") N2 (intervention* or program* or condition or "non professional*" or nonprofessional* or paraprofessional* or volunteer*)) ) | 1,550 |
| S29 | TI ( ((outreach or support or family) W0 worker*) ) OR AB ( ((outreach or support or family) W0 worker*) ) | 2,286 |
| S28 | TI ( ("village worker*" or "village health* worker*" or "village health care worker*") ) OR AB ( ("village worker*" or "village health* worker*" or "village health care worker*") ) | 140 |
| S27 | TI ( (("non professional*" or nonprofessional* or paraprofessional*) N2 (counselor* or counsellor* or counseling or counselling or coach* or intervention* or support or outreach or delivered or staff or led or provider* or based or volunteer* or mentor* or educator* or visitor* or adviser* or advisor* or facilitator* or personnel)) ) OR AB ( (("non professional*" or nonprofessional* or paraprofessional*) N2 (counselor* or counsellor* or counseling or counselling or coach* or intervention* or support or outreach or delivered or staff or led or provider* or based or volunteer* or mentor* or educator* or visitor* or adviser* or advisor* or facilitator* or personnel)) ) | 470 |
| S26 | TI ( (volunteer* N2 (counselor* or counsellor* or counseling or counselling or coach* or intervention* or support or outreach or delivered or staff or led or provider* or based or mentor* or educator* or visitor* or adviser* or advisor*)) ) OR AB ( (volunteer* N2 (counselor* or counsellor* or counseling or counselling or coach* or intervention* or support or outreach or delivered or staff or led or provider* or based or mentor* or educator* or visitor* or adviser* or advisor*)) ) | 2,549 |
| S25 | TI ( (peer W0 (counselor* or counsellor* or counseling or counselling or coach* or intervention* or support or outreach or delivered or staff or led or provider* or based or volunteer* or mentor* or educator* or visitor* or adviser* or advisor* or facilitator* or personnel)) ) OR AB ( (peer W0 (counselor* or counsellor* or counseling or counselling or coach* or intervention* or support or outreach or delivered or staff or led or provider* or based or volunteer* or mentor* or educator* or visitor* or adviser* or advisor* or facilitator* or personnel)) ) | 9,946 |
| S24 | TI ( ((peer W0 worker*) or (peer W0 "health* worker*") or (peer W0 "health care worker*")) ) OR AB ( ((peer W0 worker*) or (peer W0 "health* worker*") or (peer W0 "health care worker*")) ) | 193 |
| S23 | TI ( ("village based worker*" or "village based health* worker*" or "village based health care worker*") ) OR AB ( ("village based worker*" or "village based health* worker*" or "village based health care worker*") ) |  |
| S22 | TI ( ((lay w0 worker*) or (lay W0 "health* worker*") or (lay W0 "health care worker*")) ) OR AB ( ((lay w0 worker*) or (lay W0 "health* worker*") or (lay W0 "health care worker*")) ) | 359 |
| S21 | TI ( (lay N2 (counsellor* or counsellor* or counseling or counselling or coach* or intervention* or support or outreach or delivered or staff or led or provider* or based or volunteer* or mentor* or educator* or visitor* or adviser* or advisor* or facilitator* or person*)) ) OR AB ( (lay N2 (counsellor* or counsellor* or counseling or counselling or coach* or intervention* or support or outreach or delivered or staff or led or provider* or based or volunteer* or mentor* or educator* or visitor* or adviser* or advisor* or facilitator* or person*)) ) | 1,636 |
| S20 | TI ( ("community based worker*" or "community based health* worker*" or "community based health care worker*" or "community based volunteer*") ) OR AB ( ("community based worker*" or "community based health* worker*" or "community based health care worker*" or "community based volunteer*") ) | 183 |
| S19 | TI ( ("community worker*" or "community health* worker*" or "community health care worker*" or "community volunteer*" or "non specialist*" or "nonspecialist*") ) OR AB ( ("community worker*" or "community health* worker*" or "community health care worker*" or "community volunteer*" or "non specialist*" or "nonspecialist*") ) | 5,869 |
| S18 | TI ( ((care or aide or aides or nursing or support or intervention* or treatment* or visit*) N2 (lay or volunteer* or voluntary)) ) OR AB ( ((care or aide or aides or nursing or support or intervention* or treatment* or visit*) N2 (lay or volunteer* or voluntary)) ) | 3,626 |
| S17 | TI ( (home W0 (care or aide or aides or nursing or support or intervention* or treatmen* or visit*)) ) OR AB ( (home W0 (care or aide or aides or nursing or support or intervention* or treatmen* or visit*)) ) | 26,827 |
| S16 | TI "barefoot doctors" OR AB "barefoot doctors" | 22 |
| S15 | TI ( (linkworker* or link worker*) ) OR AB ( (linkworker* or link worker*) ) | 1,573 |
| S14 | TI ( (church based N2 (intervention* or program* or counsel*)) ) OR AB ( (church based N2 (intervention* or program* or counsel*)) ) | 409 |
| S13 | TI ( (peer w0 (volunteer* or counsel* or support or intervention*)) ) OR AB ( (peer w0 (volunteer* or counsel* or support or intervention*)) ) | 6,709 |
| S12 | TI ( (community N2 (volunteer* or aide or aides or support)) ) OR AB ( (community N2 (volunteer* or aide or aides or support)) ) | 8,537 |
| S11 | TI ( ((community or village*) N2 (health worker* or health care worker* or healthcare worker*)) ) OR AB ( ((community or village*) N2 (health worker* or health care worker* or healthcare worker*)) ) | 7,062 |
| S10 | TI ( (trained N2 (volunteer* or health worker*)) ) OR AB ( (trained N2 (volunteer* or health worker*)) ) | 1,465 |
| S9 | TI ( (lay health worker* or lay volunteer*) ) OR AB ( (lay health worker* or lay volunteer*) ) | 915 |
| S8 | TI ( ((lay or voluntary or volunteer* or untrained or unlicensed or nonprofessional* or “non professional*”) N4 (worker* or visitor* or attendant* or aide or aides or support* or person* or helper* or carer* or caregiver* or care giver* or consultant* or assistant* or visit*)) ) OR AB ( ((lay or voluntary or volunteer* or untrained or unlicensed or nonprofessional* or “non professional*”) N4 (worker* or visitor* or attendant* or aide or aides or support* or person* or helper* or carer* or caregiver* or care giver* or consultant* or assistant* or visit*)) ) | 5571 |
| S7 | social support | 137,701 |
| S6 | peer group or peer support or peer counseling or support group | 173,566 |
| S5 | home nursing | 78,417 |
| S4 | volunteers or volunteering or volunteer work or volunteer | 56,154 |
| S3 | homemaker services | 536 |
| S2 | home health aides | 2,294 |
| S1 | community health workers | 19,100 |

Search Name: BA AND LAY WORKERS V1 11-121-2024

Date Run: 07/01/2025 02:52:32

Comment:

ID Search Hits

#1 MeSH descriptor: [Community Health Workers] explode all trees 853

#2 MeSH descriptor: [Home Health Aides] explode all trees 33

#3 MeSH descriptor: [Homemaker Services] explode all trees 6

#4 MeSH descriptor: [Volunteers] explode all trees 7393

#5 MeSH descriptor: [Home Nursing] explode all trees 342

#6 MeSH descriptor: [Health Personnel] this term only 2049

#7 MeSH descriptor: [Peer Group] explode all trees 2095

#8 ((lay or voluntary or volunteer* or untrained or unlicensed or nonprofessional* or non NEXT professional) near/5 (worker* or visitor* or attendant* or aide or aides or support* or person* or helper* or carer* or caregiver* or care NEXT giver or consultant* or assistant* or visit*)):ti,ab,kw 2825

#9 ((lay NEXT health worker* or lay NEXT volunteer*)):ti,ab,kw 330

#10 ((trained near/3 (volunteer* or health NEXT worker*))):ti,ab,kw 854

#11 ((village NEXT worker* or village NEXT health* worker or village NEXT health care worker*)):ti,ab,kw 245

#12 (((community or village*) near/3 (health NEXT worker* or health NEXT care worker* or healthcare NEXT worker*))):ti,ab,kw 3237

#13 ((community near/3 (volunteer* or aide or aides or support))):ti,ab,kw 1632

#14 (peer near/0 (volunteer* or counsel* or support or intervention*)) 5

#15 ((church NEXT based near/3 (intervention* or program* or counsel*))):ti,ab,kw 88

#16 ((linkworker* or link NEXT worker)):ti,ab,kw 21

#17 (barefoot NEXT doctor*):ti,ab,kw 0

#18 ((home near/0 (care or aide or aides or nursing or support or intervention* or treatmen* or visit*))):ti,ab,kw 18

#19 (((care or aide or aides or nursing or support or intervention* or treatment* or visit*) near/3 (lay or volunteer* or voluntary))):ti,ab,kw 3787

#20 ((community NEXT worker or community NEXT health* worker* or community NEXT health care worker* or community NEXT volunteer* or non NEXT specialist* or nonspecialist*)):ti,ab,kw 9035

#21 ((community NEXT based worker or community NEXT based health* worker or community NEXT based health care worker or community NEXT based volunteer)):ti,ab,kw 18566

#22 ((lay near/3 (counsellor* or counsellor* or counseling or counselling or coach* or intervention* or support or outreach or delivered or staff or led or provider* or based or volunteer* or mentor* or educator* or visitor* or adviser* or advisor* or facilitator* or person*))):ti,ab,kw 957

#23 (((lay near worker*) or (lay near health* worker*) or (lay near health care worker*))):ti,ab,kw 412

#24 ((village NEXT based worker* or village NEXT based health* worker* or village NEXT based health care worker*)):ti,ab,kw 304

#25 (((peer near worker*) or (peer near health* worker*) or (peer near health NEXT care worker))):ti,ab,kw 310

#26 ((peer near (counselor* or counsellor* or counseling or counselling or coach* or intervention* or support or outreach or delivered or staff or led or provider* or based or volunteer* or mentor* or educator* or visitor* or adviser* or advisor* or facilitator* or personnel))):ti,ab,kw 5979

#27 ((volunteer* near/3 (counselor* or counsellor* or counseling or counselling or coach* or intervention* or support or outreach or delivered or staff or led or provider* or based or mentor* or educator* or visitor* or adviser* or advisor*))):ti,ab,kw 2126

#28 (((non NEXT professional* or nonprofessional* or paraprofessional*) near/3 (counselor* or counsellor* or counseling or counselling or coach* or intervention* or support or outreach or delivered or staff or led or provider* or based or volunteer* or mentor* or educator* or visitor* or adviser* or advisor* or facilitator* or personnel))):ti,ab,kw 241

#29 ((village NEXT worker* or village NEXT health* worker or village NEXT health care worker*)):ti,ab,kw 245

#30 (((outreach or support or family) near worker*)):ti,ab,kw 1153

#31 (((home NEXT visit or household NEXT visit) near/3 (intervention* or program* or condition or non NEXT professional* or nonprofessional* or paraprofessional* or volunteer*))):ti,ab,kw 289

#32 {OR #1-#31} 42548

#33 MeSH descriptor: [Depression] this term only 19070

#34 MeSH descriptor: [Mood Disorders] this term only 1166

#35 MeSH descriptor: [Depressive Disorder] this term only 9985

#36 MeSH descriptor: [Depressive Disorder, Major] this term only 7390

#37 MeSH descriptor: [Depressive Disorder, Treatment-Resistant] this term only 793

#38 MeSH descriptor: [Dysthymic Disorder] explode all trees 203

#39 MeSH descriptor: [Cyclothymic Disorder] explode all trees 25

#40 ((depress* or dysthymi* or cyclothymi* or low NEXT mood or mood NEXT disorder* or affective NEXT disorder*)):ti,ab,kw 126455

#41 {OR #33-#40} 126455

#42 #32 AND #41 4552

#43 MeSH descriptor: [Behavior Therapy] explode all trees 25815

#44 (behavio* NEXT activat*) 1649

#45 ((behavio* NEXT activat* or BATD)) 1654

#46 ((behavio* near/3 (reinforce* or re NEXT inforce*))) 610

#47 ((behavio* near/2 (contracting or modif*))) 3217

#48 (reinforc*) 12643

#49 (((positive near/1 reinforc*) or (reinforc* near/3 (environment* or experience*)))) 627

#50 (behavio* near/3 motivat*) 3047

#51 ((activit* near/2 schedul*)) 461

#52 ((pleas* or enjoyable or rewarding) near/3 (activit* or event?)) 707

#53 ((operant or instrumental) near/1 (conditioning or learning)) 489

#54 (("positive NEXT interaction" or avoida* coping or environmental NEXT contingenc* or "contingency management")) 2090

#55 ("functional analysis") 353

#56 (behavio* (self near/1 (evaluat* or monitor*))) 4088

#57 (behavio* near/1 (counsel* or intervention* or train* or treatment* or therap* or psychotherap*)) 46847

#58 ((mood near/3 monitor*)) 298

#59 {OR #43-#58} 74117

#60 #42 AND #59 1036

**WHO clinical trials (n=10 findings)**

**Search terms:** Community Workers or lay worker AND behavio*

<https://trialsearch.who.int/AdvSearch.aspx>

**----------------------------------------------------------------------------------------------------------------**

**Clinical trials (n=16)**

**Other terms:** lay worker or lay workers or community workers or community worker

AND behavior therapy OR behaviour therapy OR behavioural activation

<https://clinicaltrials.gov/search?term=lay%20worker%20or%20lay%20workers%20or%20community%20workers%20or%20community%20worker&intr=behavior%20therapy%20OR%20behaviour%20therapy%20OR%20behavioural%20activation%20&page=2>

**ANZCTR** (**13 findings)**

Key words

behavio*

Treatment other,

behavioural,

depression,

<https://www.anzctr.org.au/TrialSearch.aspx#&&conditionCode=&dateOfRegistrationFrom=&interventionDescription=behavio*&interventionCodeOperator=OR&primarySponsorType=&gender=&distance=&postcode=&pageSize=20&ageGroup=&recruitmentCountryOperator=OR&recruitmentRegion=&ethicsReview=&countryOfRecruitment=&registry=&searchTxt=&studyType=&allocationToIntervention=&dateOfRegistrationTo=&recruitmentStatus=&interventionCode=Treatment%3a+Other&healthCondition=depression&healthyVolunteers=&page=1&conditionCategory=Public+Health&fundingSource=&trialStartDateTo=&trialStartDateFrom=&phase=>

**GOOGLE SCHOLAR**

lay or voluntary or volunteer or untrained or unlicensed or non professional worker AND Behavior Therapy OR Behavioural activation OR functional analysis OR positive interaction

**5890 results**

**Download only first 10 pages (100)**
